# Structures of the interleukin 11 signalling complex reveal gp130 dynamics and the inhibitory mechanism of a cytokine variant

**DOI:** 10.1038/s41467-023-42754-w

**Published:** 2023-11-20

**Authors:** Riley D. Metcalfe, Eric Hanssen, Ka Yee Fung, Kaheina Aizel, Clara C. Kosasih, Courtney O. Zlatic, Larissa Doughty, Craig J. Morton, Andrew P. Leis, Michael W. Parker, Paul R. Gooley, Tracy L. Putoczki, Michael D. W. Griffin

**Affiliations:** 1 Department of Biochemistry and Pharmacology, Bio21 Molecular Science and Biotechnology Institute, University of Melbourne, Parkville, Victoria, 3010 Australia; 2 Ian Holmes Imaging Centre, Bio21 Molecular Science and Biotechnology Institute, University of Melbourne, Parkville, Victoria, 3010 Australia; 3 ARC Centre for Cryo-electron Microscopy of Membrane Proteins, Bio21 Molecular Science and Biotechnology Institute, University of Melbourne, Parkville, Victoria, 3010 Australia; 4 Walter and Eliza Hall Institute of Medical Research, Parkville, Victoria, 3052 Australia; 5 Department of Medical Biology, University of Melbourne, Parkville, Victoria, 3010 Australia; 6 St Vincent’s Institute of Medical Research, Fitzroy, Victoria, 3065 Australia; 7 Present Address: Center for Structural Biology, Center for Cancer Research, National Cancer Institute, Frederick, Maryland, 21702 USA; 8 Present Address: CSIRO Biomedical Manufacturing Program, Clayton, Victoria, 3168 Australia

**Keywords:** Cell signalling, Interleukins, Cancer, Cryoelectron microscopy, X-ray crystallography

## Abstract

Interleukin (IL-)11, an IL-6 family cytokine, has pivotal roles in autoimmune diseases, fibrotic complications, and solid cancers. Despite intense therapeutic targeting efforts, structural understanding of IL-11 signalling and mechanistic insights into current inhibitors are lacking. Here we present cryo-EM and crystal structures of the human IL-11 signalling complex, including the complex containing the complete extracellular domains of the shared IL-6 family β-receptor, gp130. We show that complex formation requires conformational reorganisation of IL-11 and that the membrane-proximal domains of gp130 are dynamic. We demonstrate that the cytokine mutant, IL-11 Mutein, competitively inhibits signalling in human cell lines. Structural shifts in IL-11 Mutein underlie inhibition by altering cytokine binding interactions at all three receptor-engaging sites and abrogating the final gp130 binding step. Our results reveal the structural basis of IL-11 signalling, define the molecular mechanisms of an inhibitor, and advance understanding of gp130-containing receptor complexes, with potential applications in therapeutic development.

## Abstract (web summary)

Interleukin (IL-)11 is a signaling protein that plays key roles in several human diseases. Here the authors describe structures of IL-11 in complex with its receptors and determine the molecular mechanisms through which a variant of IL-11 inhibits signalling, opening avenues for therapeutic development.

## Introduction

Interleukin (IL-)11 is secreted by numerous immune cells including CD8 + T-cells, B-cells, macrophages, natural killer (NK) cells, γδT cells, and eosinophils, and has been implicated in the differentiation of B-cells, T-cells, and anti-tumour immune responses^1–5^. IL-11 is also produced by inflammatory fibroblasts and epithelial cells resulting in a coordinated wound response to enable the maintenance of mucosal barriers^6–11^. It is now appreciated that the biological function of IL-11 spans beyond its classical role in megakaryocytopoiesis^12,13^, where recombinant IL-11 (Neumega) is FDA approved to support platelet reconstitution following chemotherapy^14^. Critical pathological roles for dysregulated IL-11 have been identified in autoimmune diseases including arthritis, asthma, inflammatory bowel disease, multiple sclerosis, and systemic sclerosis^3,4,6,7,15,16^. In addition, IL-11 drives fibrotic complications of the gastrointestinal tract, heart, kidney, liver and lung^6,9–11,17–20^, and promotes the growth of several malignancies, including breast, lung, endometrial and gastrointestinal cancers^21–25^. Despite these physiological and pathological roles, structural understanding of IL-11 signalling has remained limited.

IL-11 activates downstream signalling pathways by binding its two cell surface receptors: the IL-11 specific receptor, IL-11Rα, and the signal-transducing receptor, glycoprotein (gp)130^13,26^. Following the formation of the receptor complex, the Janus kinase (JAK)/signal transducer and activator of stat (STAT) and extracellular signal regulated kinase (ERK)/mitogen-activated protein kinase (MAPK) pathways are primarily activated^13,27–29^. IL-11 is a member of the IL-6 family of cytokines, which also includes IL-6, IL-27, IL-31, IL-35, IL-39 leukaemia inhibitory factor (LIF), oncostatin M (OSM), ciliary neurotrophic factor (CNTF), cardiotrophin-1 (CT-1), cardiotrophin-like cytokine factor 1 (CLCF1), and an analogue of IL-6 from human herpes virus 8 (vIL-6)^30–32^. The IL-6 family is commonly defined by the shared use of gp130, and thus overlaps with the IL-12 family^33^. IL-11 and IL-6 utilise a homodimer of gp130^26,34,35^ while other IL-6 family members exploit a heterodimer of gp130 with a second signal-transducing receptor, such as LIF receptor (LIFR), OSM receptor (OSMR), IL-27R/WSX-1 or IL-12 receptor β−2 subunit (IL-12Rβ2)^36^. IL-31 is unique in its use of the gp130-like receptor chain, IL-31RA, and OSMR. gp130 is a member of the tall type-I cytokine receptor family, with a large extracellular region comprising six domains, D1-D6^27^. Crystal structures of IL-6^37^, vIL-6^34^, LIF^37^ and IL-27^38,39^ in complex with D1-D3 of gp130, in addition to cryo-EM structures of CNTF, CLCF1, LIF, IL-27, and IL-6 in complex with their receptor ectodomains^40^, show that the membrane distal domains are involved in cytokine binding. However, the nature of the membrane proximal D4-D6 domains of gp130 within cytokine-receptor complexes has not been fully elucidated^40^.

Inhibition of IL-11 signalling has been shown to provide therapeutic benefit in models of arthritis, multiple sclerosis, neointimal hyperplasia, multiple fibrotic diseases, and gastrointestinal cancers^9–11,13,17,20,21,24,41,42^. Current IL-11 signalling inhibitors include IL-11 mutants^43,44^ and antibodies against either IL-11^9,20,45^ or IL-11Rα^11,46–48^. However, mechanistic understanding of their modes of action is limited in the absence of detailed molecular understanding of the IL-11 signalling complex. Informed development of new and existing signalling inhibitors requires a comprehensive understanding of the structure and assembly mechanisms of the IL-11 signalling complex.

Here, we present structures of the human IL-11 signalling complex, providing detail of the molecular mechanisms of complex formation and the structure and dynamics of the complete extracellular domains of gp130 within the complex. We characterise an IL-11 variant, IL-11 Mutein, that potently inhibits IL-11 signalling and describe the detailed mechanism of its action. Our results validate IL-11 Mutein as a tool to inhibit IL-11 signalling and provide a straightforward method for its production. Our insights reveal the structural basis of IL-11 signalling and provide invaluable molecular platforms for development of existing and novel therapeutics targeting IL-11 signalling and other class I cytokines.

## Results

### The structure of the IL-11 signalling complex

**Fig. 1:**
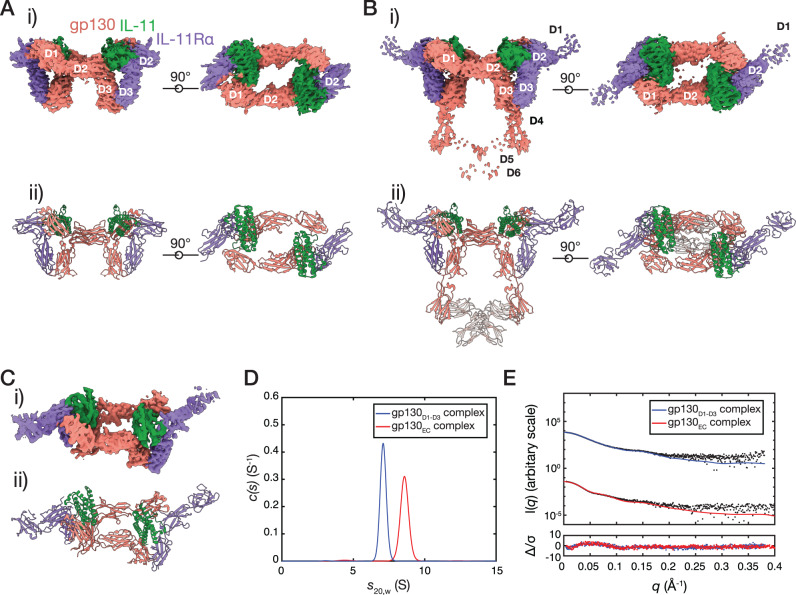
*Structure of the IL-11 signalling complex* (IL-11, green; IL-11Rα, purple; gp130 salmon). **A** Cryo-EM density map (contoured at 7 σ), (i), and atomic model, (ii), of the IL-11_Δ10_/IL-11Rα_D1-D3_/gp130_D1-D3_ complex. D1 of IL-11Rα was not modelled in this structure. **B** Cryo-EM density map (contoured at 7 σ), (i) and atomic model, (ii), of the IL-11_Δ10_/IL-11Rα_D1-D3_/gp130_EC_ complex. The position of the D5-D6 domains of gp130 is indicated as transparent ribbons. These domains were not included in the deposited model. Individual domains of IL-11Rα and gp130 are indicated on the cryo-EM maps. **C** X-ray electron density (contoured at 1 σ, with missing *F*_obs_ not filled), (i), and crystal structure of the IL-11_FL_/IL-11Rα_EC_/gp130_D1-D3_ complex, (ii), showing one hexamer of the asymmetric unit. **D** Continuous sedimentation coefficient (c(s)) distributions for the IL-11_Δ10_/IL-11Rα_D1-D3_/gp130_D1-D3_ complex and the IL-11_Δ10_/IL-11Rα_D1-D3_/gp130_EC_ complex. **E** SAXS data for the IL-11_Δ10_/IL-11Rα_D1-D3_/gp130_D1-D3_ complex and the IL-11_Δ10_/IL-11Rα_D1-D3_/gp130_EC_ complex.

To understand the molecular mechanisms underpinning human IL-11 signalling complex formation, we solved three structures of the complex (Fig. 1) containing either the cytokine binding domains of gp130 (gp130_D1-D3_) or the complete extracellular domains of gp130 (gp130_EC_) using electron cryo-microscopy (cryo-EM) and X-ray crystallography. Complexes included an N-terminally truncated form of IL-11 (IL-11_Δ10_) or full-length IL-11 (IL-11_FL_), and a C-terminally truncated form of IL-11Rα (IL-11Rα_D1-D3_) or the complete extracellular domains of IL-11Rα (IL-11Rα_EC_) described previously^49^.

We obtained a 3.5 Å resolution cryo-EM reconstruction of the IL-11_Δ10_/IL-11Rα_D1-D3_/gp130_D1-D3_ complex (referred to as the gp130_D1-D3_ complex) (Fig. 1Ai; Supplementary Fig. 1A; Supplementary Fig. 2A, B; Supplementary Table 1), and a 3.8 Å resolution cryo-EM reconstruction of the IL-11_Δ10_/IL-11Rα_D1-D3_/gp130_EC_ complex (referred to as the gp130_EC_ complex) (Fig. 1Bi, Supplementary Fig. 1B; Supplementary Fig. 2C, D; Supplementary Table 1). We used these data to build and refine the atomic models of the IL-11 signalling complex (Fig. 1Aii, Bii). We also obtained crystals of the IL-11_FL_/IL-11Rα_EC_/gp130_D1-D3_ complex that diffracted anisotropically to 3.8 Å. We used the cryo-EM structure of the gp130_D1-D3_ complex to phase the X-ray diffraction data by molecular replacement (Fig. 1C). Three IL-11 signalling complexes were arranged in a triangular configuration in the asymmetric unit providing 6-fold non-crystallographic symmetry to aid refinement (Supplementary Fig. 3A–C).

Secondary structure was clearly visible in both cryo-EM density maps, larger side chains were generally defined, the α-helical structure of the cytokine was clear, and β-strands were generally separated, consistent with maps reconstructed at these resolutions (Supplementary Fig. 2B, D). D1 of IL-11Rα was not visible in the gp130_D1-D3_ complex map and was poorly defined in the gp130_EC_ complex map (Supplementary Fig. 2Di), suggesting this domain is flexible. Satisfactory density defining the positions of the β-sheets of IL-11Rα D1 facilitated refinement of the domain in the gp130_EC_ complex and the crystal structure, confirming the average positions of this domain in the complex (Supplementary Fig. 2Di; Supplementary Fig. 3). N-linked glycans were visible on IL-11Rα (N105, N172) and gp130 (N21, N61, N135) in all structures (Supplementary Fig. 4). D5-D6 of gp130 were poorly defined in the gp130_EC_ complex density (Fig. 1Bi) and were not included in the deposited model. However, density was sufficient to refine the positions of these domains as rigid bodies (shown in grey in Fig. 1Bii).

All structures show a hexamer consisting of two copies each of IL-11, IL-11Rα and gp130, in agreement with previous immunoprecipitation and native-gel electrophoresis experiments^26^, and a low-resolution EM map of the complex^50^. The structures bear a striking resemblance to a table, with D2 of IL-11Rα and gp130, and IL-11 forming the table-top, and D3 of IL-11Rα and gp130 forming the legs (Fig. 1Aii), similar to the IL-6^35^ and vIL-6^34^ complexes. The density for the gp130_EC_ complex shows that D5-D6 of the two gp130 molecules cross over (Fig. 1Bii), as previously suggested by low-resolution EM reconstructions of the complete extracellular domains of the IL-6^51,52^ and IL-11^50^ signalling complexes, and recent cryo-EM studies of the IL-6 signalling complex^40^.

Sedimentation velocity analytical ultracentrifugation (SV-AUC) provided sedimentation coefficient distributions with single, narrow peaks for the gp130_D1-D3_ and gp130_EC_ complexes at 7.1 S and 8.4 S, respectively (Fig. 1D). Molecular masses for the gp130_D1-D3_ and gp130_EC_ complexes estimated from SV-AUC of 178.7 kDa (frictional ratio [f/f_0_]: 1.6) and 269.4 kDa (f/f_0_: 1.8) were consistent with masses calculated from sequence (169.8 kDa and 235.0 kDa) as were masses determined by multi-angle light scattering (MALS) of *M*_w_: 187.3 kDa and *M*_w_: 259.6 kDa (Supplementary Fig. 5A). Higher experimental masses than those calculated from sequence are likely due to N-linked glycans present on the receptors.

To confirm the solution stoichiometry, we used sedimentation equilibrium analytical ultracentrifugation (SE-AUC), which provides a relative molecular mass (M*) for the complex and its components independent of the extent of glycosylation. SE-AUC of the gp130_D1-D3_ and gp130_EC_ complexes yielded M* of 176.5 kDa and 255.5 kDa respectively (Supplementary Fig. 5C–E), in excellent agreement with the sum of the M* values obtained for the hexamer components of 179.2 kDa and 264.4 kDa.

To further probe the solution configuration of the hexamer we collected small-angle X-ray scattering (SAXS) data for the gp130_D1-D3_ and gp130_EC_ complexes (Fig. 1E; Supplementary Fig. 6A–D; Supplementary Table 3). Our atomic coordinates fit the scattering data well (Fig. 1E, Supplementary Table 3), and masses derived from the data of 182.2 kDa and 290.0 kDa agree with calculated hexamer masses. Deviations of the theoretical scattering profile from the experimental data at high *q* may be due to truncated N-linked glycans in the coordinates. Together these data confirm that our atomic models represent the solution configuration of the hexameric IL-11 signalling complex.

### The IL-11 signalling complex forms in three steps and requires significant structural rearrangement of the cytokine

**Fig. 2:**
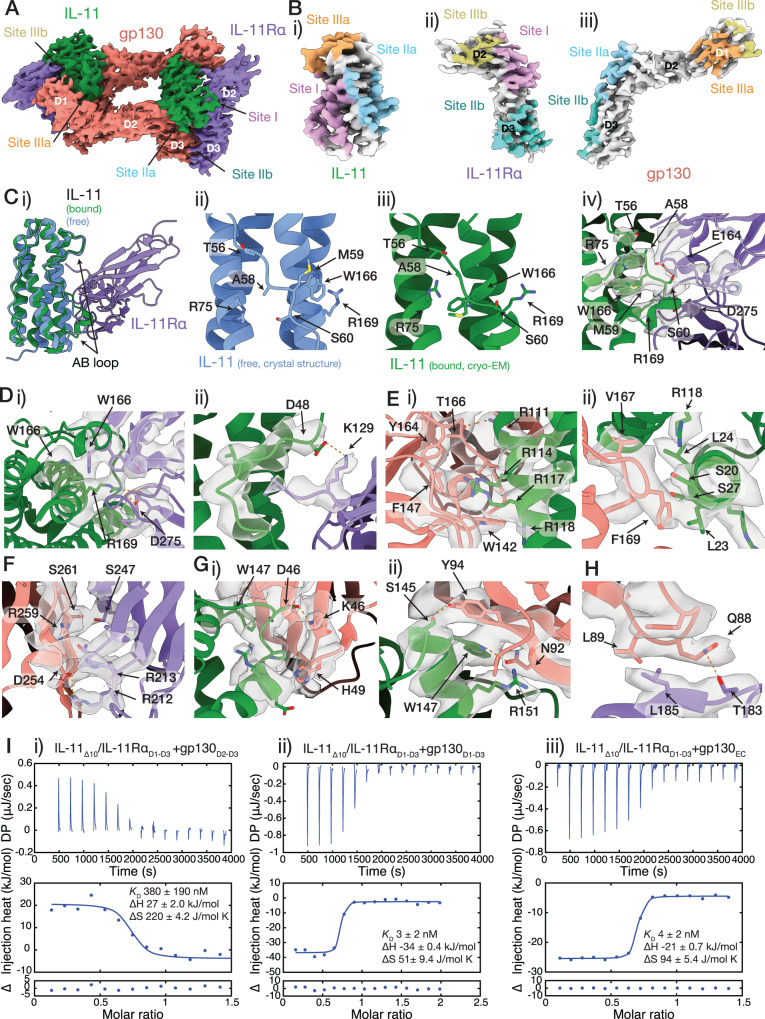
Interactions forming the IL-11 signalling complex. Bound IL-11 is depicted in green, IL-11Rα in purple, and gp130 in salmon. **A** Structure of the IL-11_Δ10_/IL-11Rα_D1-D3_/gp130_D1-D3_ complex, with the five binding sites indicated. **B** Binding surfaces on (i) IL-11, (ii) IL-11Rα, and (iii) gp130. **C** Rearrangement of the AB loop on complex formation. Uncomplexed IL-11 is depicted in blue. (i) overlay of the crystal structure of IL-11_Δ10_^49^ (PDB ID: 6O4O) with the structure of the complex, showing the AB loop (F43-G65) rearrangement on complex formation. (ii) interactions within the AB loop, and between the AB loop and the α-helical core in the unbound state. (iii) and (iv) the AB loop rearrangement on complex formation. **D** Details of site-I contacts (IL-11, green; IL-11Rα, purple; gp130 salmon). (i) R169 of IL-11 protrudes into a pocket formed by several hydrophobic residues on IL-11Rα, (ii) contacts between the N-terminal end of the AB loop of IL-11 and IL-11Rα. **E** Details of site-IIA contacts, (i) contacts between C-helix arginine residues of IL-11 and gp130, (ii) contacts between the N-terminal end of the A helix of IL-11 and gp130. **F** Details of site-IIB contacts. **G** Details of site-IIIA contacts. (i) contacts between W147 and the N-terminal end of the AB loop of IL-11 with D1 of gp130. (ii) contacts between W147 and neighbouring residues of IL-11 with D1 of gp130. **H** Details of site-IIIB contacts. Cryo-EM density maps are contoured at 7 σ. **I** Representative ITC data for (i) the interaction between the IL-11_Δ10_/IL-11Rα_D1-D3_ binary complex and gp130_D2-D3_; (ii) the interaction between the IL-11_Δ10_/IL-11Rα_D1-D3_ binary complex and gp130_D1-D3_; and (iii), the interaction between the IL-11_Δ10_/IL-11Rα_D1-D3_ binary complex and gp130_EC_. Representative of *n* = 3 independent experiments. For complete thermodynamic parameters, see Supplementary Table 4.

Our structures of the IL-11 signalling complex allow precise identification of the interactions forming the complex (Fig. 2A, B). Overall, complex formation results in the burying of ~6450 Å^2^ of surface area. The binding sites on IL-11 (Fig. 2B) contain residues previously identified by mutagenesis of human and mouse IL-11^53–55^ and by modelling^49^. The structures reveal additional contacts between the loop joining helices A and B of the cytokine (AB loop; residues F43-G65) and IL-11Rα in site-I and gp130 in site-III that have not previously been identified or interrogated (Fig. 2C, D).

Initial formation of a 1:1 complex between IL-11 and IL-11Rα is mediated through site-I of the cytokine (Fig. 2C, D). The interaction has an affinity of 23 nM^49^ and is strongly driven by entropy, burying ~1000 Å^2^ of surface area. The C-terminal section of the AB loop undergoes a rearrangement upon complex formation, relative to uncomplexed IL-11^49^ (Fig. 2C), resulting in significant conformational change of residues 58-68, including unfolding of the N-terminal turn of helix B. A cation-π interaction between R169 and W166 in uncomplexed IL-11^49^ is broken on complex formation, allowing the AB loop to adopt a new conformation and form extensive contacts with IL-11Rα (Fig. 2Cii–iv). The rearrangement allows R169 of IL-11 to protrude into a hydrophobic pocket formed by F165, F230, and L277 of IL-11Rα, and to form a potential salt bridge with D275 (Fig. 2Civ, Di). S60 and the backbone amide of M59 within the AB loop both form potential hydrogen bond networks with E164 of IL-11Rα after rearrangement of the AB loop. The key roles of R169 of IL-11 in mediating both contacts with IL-11Rα and conformational change of the AB loop and helix B are consistent with the significant reduction in affinity of IL-11 for IL-11Rα upon substitution of R169 with alanine^49^. Notably, the new position of the AB loop is stabilised by several new intra-IL-11 contacts, including a potential hydrogen bond formed by the δ-sulfur of M59 and the indole nitrogen of W166, and two potential hydrogen bonds formed by the guanidinium group of R75 with backbone atoms of T56 and A58 in the AB loop (Fig. 2Ciii). Several additional contacts are formed by the N-terminal end of the AB loop, including a potential salt bridge between D48 on the cytokine and K129 on IL-11Rα (Fig. 2Dii). However, these contacts do not result in conformational change in this region of the cytokine.

After binding IL-11Rα, IL-11 engages two molecules of gp130, via site-II and site-III, to form the hexameric signalling complex (Fig. 2E–H). Site-III interactions are mediated by D1 of gp130 suggesting sequential complex assembly via site-II followed by site-III. To probe the intermediate in IL-11 complex assembly, we generated gp130_D2-D3_, which lacks D1. SV-AUC of the IL-11_Δ10_/IL-11Rα_D1-D3_/gp130_D2-D3_ complex (gp130_D2-D3_ complex) yields a single species with sedimentation coefficient of 4.0 S, and molecular weight of 78.2 kDa (f/f_0_: 1.6; calculated Mw: 73.5 kDa), suggesting a trimeric stoichiometry (Supplementary Fig. 5F, G). MALS further supports a trimeric stoichiometry (*M*_w_ = 70.6 kDa, Supplementary Fig. 5A). SAXS data for the gp130_D2-D3_ complex fit well to a model of the trimer comprising one molecule of each component assembled via site-I and site-II (Supplementary Fig. 6B, E, Supplementary Table 3), further indicating that this trimeric complex is a stable intermediate in the complex assembly.

The interaction between the binary IL-11/IL-11Rα complex and the first molecule of gp130 comprises two coupled interfaces: (1) between IL-11 and gp130 (site-IIA, Fig. 2E), and (2) between IL-11Rα and gp130 (site-IIB, Fig. 2F). The interactions bury ~700 Å^2^ and 600 Å^2^ respectively. The site-IIA interface is hydrophobic in character and the main contact area between IL-11 and gp130 is located on helices A and C of IL-11. Important contacts are mediated by four arginine residues of IL-11 (111, 114, 117 and 118) and two loops of gp130 (residues 142-147 and 164-172) (Fig. 2Ei). Key contacts are formed by R114, which protrudes into an aromatic pocket formed by W142 and F147 of gp130. Residues W142 and F169 of gp130 also form important interactions. F169 interacts with the central section of helix A of IL-11, forming hydrophobic contacts with L23 and L24 (Fig. 2Eii). F196 of gp130 has been shown to also form key contacts with other IL-6 family cytokines^56,57^. The site-IIB interaction is electrostatic in nature, and results in the formation of ten potential hydrogen bonds between D3 of IL-11Rα and D3 of gp130 (Fig. 2F).

The final interaction to form the complex is a coupled interaction between IL-11 and gp130 D1 (site-IIIA) (Fig. 2G) and between IL-11Rα D2 and gp130 D1 (site-IIIB) (Fig. 2H). The interaction buries ~800 Å^2^ in surface area; ~600 Å^2^ at site-IIIA, and 200 Å^2^ at site-IIIB. Assembly of the hexamer occurs via two symmetrical site III interfaces between the two IL-11/IL-11Rα/gp130 trimers. The primary contact within site-IIIA is formed by the side chain of W147 of IL-11, which binds flat against a β-strand of gp130 D1 (Fig. 2G), with the indole nitrogen forming a potential hydrogen bond to N92 (Fig. 2Gi). Contacts are also formed by R151 and S145 of IL-11, which are adjacent to W147 (Fig. 2Gi). A second set of interactions are formed by the N-terminal end of the IL-11 AB loop, where P44 packs against D1 of gp130, and D46 forms hydrogen bonds with K46 and N82 of gp130 (Fig. 2Gii). A small interface is formed at site-IIIB between a β-sheet (residues 183-187) of IL-11Rα D2 and a loop of gp130 D1 (residues 86-89) (Fig. 2H). L185 of IL-11Rα forms hydrophobic contacts with L89 of gp130 and a potential hydrogen bond is formed between Q88 of gp130 and T183 of IL-11Rα (Fig. 2H).

We used isothermal titration calorimetry (ITC) to study the thermodynamics of complex assembly (Fig. 2I; Supplementary Table 4). The interaction of the IL-11_Δ10_/IL-11Rα_D1-D3_ binary complex with gp130_D2-D3_ to form the trimer is endothermic, has moderate affinity (*K*_D_ 380 ± 190 nM, ΔH 27 ± 2 kJ/mol; Fig. 2Ii), and is strongly driven by entropy. The interaction between the IL-11_Δ10_/IL-11Rα_D1-D3_ complex and gp130_D1-D3_, which includes symmetrical interactions of both molecules of gp130 within the hexamer, is exothermic and relatively high affinity (*K*_D_ 3 ± 2 nM, ΔH -34 ± 0.4 kJ/mol; Fig. 2Iii).

The free energies of binding of the IL-11_Δ10_/IL-11Rα_D1-D3_ binary complex with gp130_D2-D3_ (ΔG -36 ± 1.7 kJ/mol) and gp130_D1-D3_ (ΔG -49 ± 3.0 kJ/mol) indicate that the ΔG associated with each gp130 D1 binding at site-III is ~−7 kJ/mol. Thus, the affinity of a single gp130 molecule at site-III is low (*K*_D_ ~ mM) and it is the avidity of the symmetrically duplicated site-III interactions that maintains hexameric complex formation. The affinity of the IL-11Rα_D1-D3_/IL-11_Δ10_ binary complex for gp130_EC_, (*K*_D_ 4 ± 2 nM, ΔH -20 ± 1 kJ/mol; Fig. 2Iiii) is very similar to the affinity for gp130_D1-D3_, indicating that the membrane-proximal (D4-D6) domains of gp130 do not contribute substantially to complex formation.

Gp130 is shared by most members of the IL-6 family of cytokines. The structure of the hexameric IL-6 signalling complex^35^ showed site-II and site-III interactions with gp130 that are similar to those of the IL-11 complex (Supplementary Fig. 7A–E), and the LIF/gp130 complex structure comprises a comparable site-II interaction^37^ (Supplementary Fig. 7A, B). However, the molecular details of the interactions between the three cytokines differ (Supplementary Fig. 7B, C, E; see also Supplementary Discussion). Specifically, the IL-11 site-II interaction buries a larger surface area and is more electrostatic in nature, with a higher number of potential hydrogen bonds. In contrast, the IL-11 site-III interaction surface is smaller than that of IL-6 and does not involve the N-terminus of gp130.

### The membrane proximal domains of gp130 are highly dynamic

The crystal structure of the extracellular domains of gp130^58^ and low resolution electron microscopy studies of the IL-11^50^, IL-6^51,52^, and LIF^59^ signalling complexes suggest that the membrane proximal domains, D4-D6, are responsible for correctly positioning the transmembrane and intracellular domains of the two signalling receptors. This configuration is thought to orient intracellularly bound JAK molecules for activation, and deletion of any of D4-D6 renders gp130 non-functional in vitro^60,61^.

**Fig. 3:**
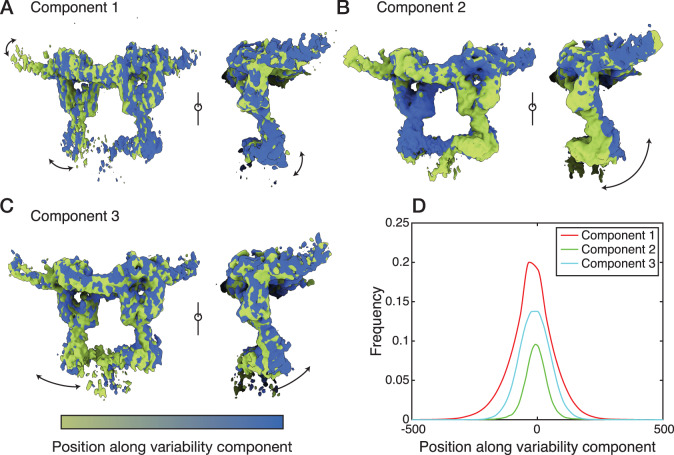
3D variability analysis of the gp130_EC_ complex cryo-EM map. Refined consensus densities along, (**A**) variability component 1, (**B**) variability component 2, (**C**) variability component 3. Density maps are coloured according to the relative position along the variability component and arrows indicate approximate motions of the receptor domains. **D** Frequency distributions of the number of particles contributing to each variability component (see also Supplementary Fig. 8).

Our consensus maps of the gp130_EC_ complex showed well-defined density for the domains involved in complex formation (D2-D3 of IL-11Rα, IL-11, and D1-D3 of gp130), and poorer density for gp130 D4-D6 (Fig. 1B; Supplementary Fig. 2Cii, Dii). We considered the possibility that structural dynamics of gp130 D4-D6 contributed to the decreasing density quality with distance from the cytokine binding regions. To explore this, we used 3D variability analysis (3DVA)^62^ in *cryoSPARC*^63^, which resolves conformational changes by fitting the multiple conformations captured in the cryo-EM particle set to a continuous linear subspace model (Fig. 3, Supplementary Fig. 8A–C). 3DVA analysis of the gp130_EC_ complex cryo-EM data reveals three major variability components (Fig. 3A–C). The first has minor motion (Fig. 3A; Supplementary Movie 1), the second corresponds to a side-to-side oscillation of D4-D6 of gp130 (Fig. 3B; Supplementary Movie 2), and the third corresponds to an inward swinging motion of D4-D6 relative to the rest of the complex (Fig. 3C, Supplementary Movie 3). Histograms of the distributions along each trajectory are unimodal, indicating continuous motion (Fig. 3D, Supplementary Fig. 8A–C). This analysis shows that the D4-D6 region of gp130 is dynamic and is not stabilised upon complex formation. A multistate model, allowing flexibility of gp130 domains D4-D6 and IL-11Rα D1, fit the SAXS data for the gp130_EC_ complex better than our single consensus model of the complex (Supplementary Fig. 8D, E; Supplementary Table 3), further supporting flexibility of these domains within the complex. The proposed flexibility of D4-D6 of gp130 is also in agreement with our ITC results, showing that the gp130_EC_ complex forms with similar affinity to the gp130_D1-D3_ complex, and is supported by our previous molecular dynamics (MD) simulations of gp130 D2-D5 showing that D4 is dynamic with respect to D3^64^.

Together these data indicate that D4-D6 of gp130 do not contribute to complex formation. Despite the separation of the C-termini of D6 in the consensus model (Supplementary Fig. 8F), the 5–6 residue linker between D6 and the transmembrane helix segment may allow interaction of the two gp130 transmembrane helices of the complex in a parallel configuration.

### The cytokine variant, IL-11 Mutein, potently inhibits IL-11 signalling in human cells

IL-11 mutants have previously been proposed to competitively inhibit IL-11 signalling^43,44^. The IL-11 W147A mutation^44^ removes the key tryptophan residue in site-III responsible for the final step of hexameric complex formation (Fig. 2Gi). Phage display was subsequently used to identify variants with higher inhibitory potency resulting in IL-11 Mutein, which contains W147A combined with mutations of AB loop residues 58–62, ^58^AMSAG^62^ to ^58^PAIDY^62^, that were reported to increase affinity to IL-11Rα^43^.

**Fig. 4:**
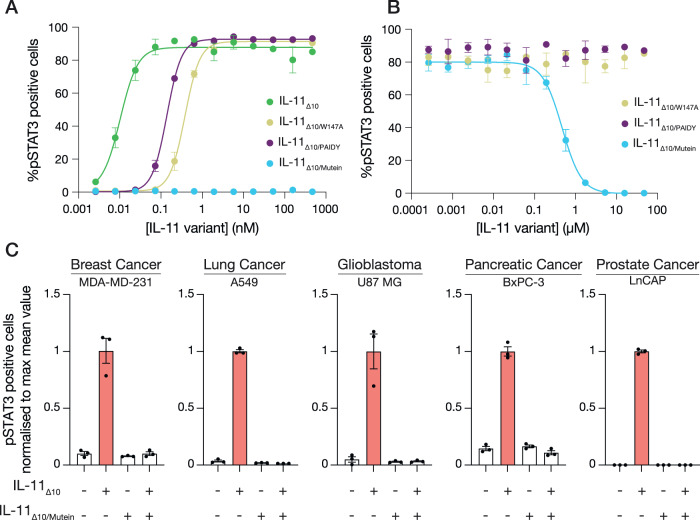
IL-11 Mutein is an effective inhibitor of human IL-11 signalling in vitro. **A** Representative dose-response curve for IL-11 or IL-11 variant stimulation. The EC_50_ for IL-11_Δ10_ was 0.010 ± 0.002 nM; the EC_50_ for IL-11_Δ10/W147A_ was 0.6 ± 0.2 nM, the EC_50_ for IL-11_Δ10/PAIDY_ was 0.14 ± 0.02 nM; the EC_50_ for IL-11_Δ10/Mutein_ could not be determined. Data are presented as the mean ± SEM of three technical replicates. Representative of *n* = 3 independent experiments. Replicate experiments are presented in Supplementary Fig. 9A. **B** Representative dose-response curve for the inhibition of IL-11_Δ10_ stimulation by the IL-11 variants IL-11_Δ10/W147A_, IL-11_Δ10/PAIDY_, and IL-11_Δ10/Mutein_. The IC_50_ for IL-11_Δ10/Mutein_ inhibition was 850 ± 275 nM, the IC_50_ for the remaining variants was not determined. Data are presented as the mean ± SEM of three technical replicates. Representative of *n* = 3 independent experiments. Replicate experiments are presented in Supplementary Fig. 9B. **C** Potent inhibition of IL-11_Δ10_ signalling by IL-11_Δ10/Mutein_ in the indicated human cancer cell lines. Data are presented as the mean ± SEM of three technical replicates. Representative of *n* = 2 independent experiments. Replicate experiments are presented in Supplementary Fig. 9C. Source data are provided as a Source Data file.

To characterise these mutants, we generated recombinant human IL-11_Δ10/W147A_, IL-11_Δ10/Mutein_, and a variant containing only the ^58^PAIDY^62^ mutations, which we termed IL-11_Δ10/PAIDY_. We assessed the biological activity of IL-11_Δ10/W147A_, IL-11_Δ10/Mutein_ and IL-11_Δ10/PAIDY_ in Ba/F3 cells stably expressing human gp130 and IL-11Rα^65^ by measuring STAT3 activation, indicated by phosphorylation at Y705 (pSTAT3), using flow cytometry (Fig. 4; Supplementary Fig. 9). As expected, stimulation with IL-11_Δ10_ produces a robust pSTAT3 response, with an EC_50_ of 12 ± 1.1 pM (Fig. 4A; Supplementary Fig. 9A). Conversely, stimulation with IL-11_Δ10/Mutein_ alone does not result in STAT3 activation (Fig. 4A; Supplementary Fig. 9A), demonstrating that the mutations eliminate the ability of IL-11_Δ10/Mutein_ to signal, in agreement with previous reports^43^. Surprisingly, stimulation with either IL-11_Δ10/W147A_ or IL-11_Δ10/PAIDY_ resulted in robust STAT3 activation, albeit with a higher EC_50_ than IL-11_Δ10_, of 610 ± 120 pM (*p* = 0.04 vs IL-11_Δ10_) and 140 ± 12 pM (*p* = 0.01 vs IL-11_Δ10_), respectively.

We also examined the inhibitory activity of IL-11_Δ10/Mutein_, IL-11_Δ10/W147A_ and IL-11_Δ10/PAIDY_ in Ba/F3 cells (Fig. 4B; Supplementary Fig. 9B). IL-11_Δ10/Mutein_ strongly inhibited IL-11_Δ10_ mediated STAT3 activation (IC_50_ = 850 ± 275 nM, Fig. 4B), validating IL-11_Δ10/Mutein_ as an effective inhibitor of IL-11 signalling. In contrast, neither IL-11_Δ10/W147A_ or IL-11_Δ10/PAIDY_ inhibited IL-11_Δ10_ mediated STAT3 activation, contradicting previous reports that the W147A mutation alone is an effective IL-11 signalling inhibitor^44,66,67^. Overall, these results show that the inhibitory effect of IL-11_Δ10/Mutein_ requires the combination of both the W147A and ^58^PAIDY^62^ mutations, with either variant alone insufficient to inhibit STAT3 phosphorylation via IL-11 signalling.

We extended these studies to several human cell lines and showed that IL-11_Δ10_ robustly stimulates STAT3 activation in the breast cancer cell line MDA-MD-231, the lung cancer cell line A549, the glioblastoma cell line U87-MG, the pancreatic cancer cell line BxPC3, and the prostate cancer cell line LnCap (Fig. 4C), in keeping with the tumorigenic function of IL-11. In each of these human cell lines, IL-11_Δ10/Mutein_ effectively inhibits IL-11 mediated STAT3 activation.

### IL-11 Mutein blocks the final step of hexamer assembly

**Fig. 5:**
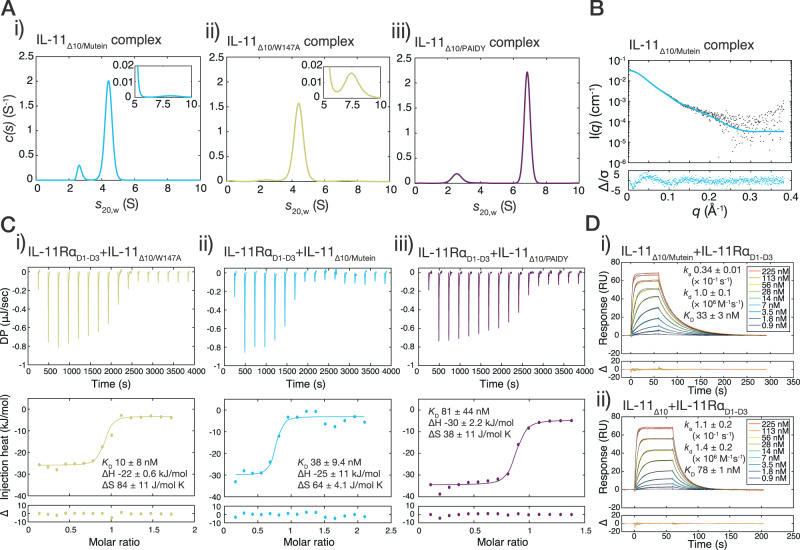
Biophysical basis for IL-11 Mutein signalling inhibition. **A** Continuous sedimentation coefficient (c(s)) distributions for the complexes formed between IL-11Rα_D1-D3_, gp130_D1-D3_ and (i) IL-11_Δ10/Mutein_, (ii) IL-11_Δ10/W147A_, (iii) IL-11_Δ10/PAIDY_. Inlays show expanded detail of the 5–10 S region. **B** SAXS data for the IL-11Rα_D1-D3_/gp130_D1-D3_/ IL-11_Δ10_ Mutein complex. The fit shown is to a model of the trimeric complex, *χ*^2^ 1.7 (see Methods). **C** Representative ITC data for the interaction between IL-11Rα_D1-D3_ and (i) IL-11_Δ10/W147A_, (ii) IL-11_Δ10/Mutein_, and (iii) IL-11_Δ10/PAIDY_. Representative of *n* = 3 independent experiments. **D** SPR data for the interaction between IL-11Rα_D1-D3_ and (i) biotinylated IL-11_Δ10/Mutein_ and (ii) biotinylated IL-11_Δ10_. Black lines show the fit to the data. Representative of *n* = 2 independent experiments. In both experiments, the biotin tag was used to immobilise IL-11_Δ10_ or IL-11_Δ10_ Mutein to a streptavidin sensor chip. For complete thermodynamic and kinetic parameters for the ITC and SPR experiments, see Supplementary Tables 4 and 5, respectively.

Our functional results prompted investigation of the structural mechanism of IL-11 Mutein signalling inhibition. SV-AUC of the IL-11_Δ10/Mutein_/IL-11Rα_D1-D3_/gp130_D1-D3_ complex yielded a single peak in the sedimentation coefficient distribution of 4.5 S and estimated mass 88.9 kDa (f/f_0_: 1.6), consistent with the formation of a homogenous trimeric complex (Fig. 5Ai; Supplementary Fig. 10Ai).

SV-AUC of the IL-11_Δ10/W147A_/IL-11Rα_D1-D3_/gp130_D1-D3_ complex indicates that the W147A mutant predominantly forms a trimeric complex (sedimentation coefficient: 4.5 S, mass: 82.7 kDa, f/f_0_: 1.5; Fig. 5Aii; Supplementary Fig. 10Aii). However, a small population of hexameric complex at ~7.5 S was observed in the sedimentation coefficient distributions, indicating that the W147A mutation alone permits some hexamer formation and consistent with the ability of IL-11_Δ10/W147A_ to stimulate STAT3 activation in vitro (Fig. 4A). SV-AUC of the IL-11_Δ10/PAIDY_/IL-11Rα_D1-D3_/gp130_D1-D3_ yields a single species with sedimentation coefficient of 6.8 S, and mass of 184.3 kDa (f/f_0_: 1.7), Fig. 5Aiii, Supplementary Fig. 10Aiii, indicating that IL-11_Δ10/PAIDY_ mediates formation of the hexameric complex to a similar extent to WT IL-11_Δ10_.

Further supporting the trimeric nature of the IL-11_Δ10/Mutein_ complex, SAXS data collected on the IL-11_Δ10/Mutein_/gp130_D1-D3_/IL-11Rα_D1-D3_ complex (Fig. 5B, Supplementary Fig. 10B) agrees well with the calculated scattering profile of a single trimer of the gp130_D1-D3_ complex. Furthermore, ab initio models from SAXS data collected on the IL-11_Δ10/Mutein_/IL-11Rα_D1-D3_/gp130_D1-D3_ complex and the gp130_D1-D3_ complex hexamer are consistent with the IL-11_Δ10/Mutein_ complex being half the size in one dimension compared to the hexameric complex (Supplementary Fig. 10C). The mass of the IL-11_Δ10/Mutein_ complex determined by SV-AUC and SAXS is also supported by the mass determined using MALS (*M*_w_ 79.6 kDa, Supplementary Fig. 10D).

In combination, these results indicate that that both IL-11_Δ10/W147A_ and IL-11_Δ10/PAIDY_ can mediate assembly of the hexameric signalling complex, albeit to differing extents, while formation of site III interactions by IL-11_Δ10/Mutein_ is abrogated, and assembly is stalled at the trimer stage.

### IL-11 Mutein/IL-11Rα binding kinetics are different to WT IL-11

The ^58^PAIDY^62^ mutations present in the AB loop of IL-11 were proposed to increase the affinity for IL-11Rα twenty-fold over WT IL-11, resulting in effective competition for IL-11Rα binding^43^. To test this, we measured the affinity of the mutant cytokines for IL-11Rα using ITC (Fig. 5C). IL-11_Δ10/W147A_, IL-11_Δ10/Mutein_, and IL-11_Δ10/PAIDY_ interact with IL-11Rα_D1-D3_ with *K*_D_ of 10 ± 8 nM, 38 ± 9 nM, (*p* = 0.2 vs IL-11_Δ10/W147A_), and 81 ± 44 nM, (*p* = 0.3 vs IL-11_Δ10/W147A_) respectively (Fig. 5C). These data suggest that the PAIDY mutations do not significantly increase the affinity of IL-11 for IL-11Rα.

We also measured binding kinetic parameters for IL-11_Δ10_ and IL-11_Δ10/Mutein_ with IL-11Rα_D1-D3_ using surface plasmon resonance (SPR). IL-11 constructs were C-terminally biotinylated via an avitag^68^ and coupled to a streptavidin SPR chip (Fig. 5D). Despite similar affinity, the kinetics of the interactions were different, with approximately three-fold slower dissociation rate for IL-11_Δ10/Mutein_/IL-11Rα_D1-D3_ (*k*_d_ 0.34 ± 0.01 × 10^−1 ^s^−1^, Fig. 5Di) than IL-11_Δ10_/IL-11Rα_D1-D3_ (*k*_d_ 1.1 ± 0.2 × 10^−1 ^s^−1^; Fig. 5Dii*n* = 2; Supplementary Table 5; *p* = 0.06 vs IL-11_Δ10/Mutein_).

These results show that the PAIDY mutations present in IL-11_Δ10/Mutein_ alter the kinetics of interaction with IL-11Rα, compared to IL-11, but do not significantly alter the affinity (Fig. 5C, *p* = 0.2 IL-11_Δ10/Mutein_ vs IL-11_Δ10/W147A_), suggesting that the mechanism of signalling inhibition by IL-11 Mutein is more complex than previously appreciated.

### The conformation of the AB loop of IL-11 Mutein is altered relative to WT IL-11

**Fig. 6:**
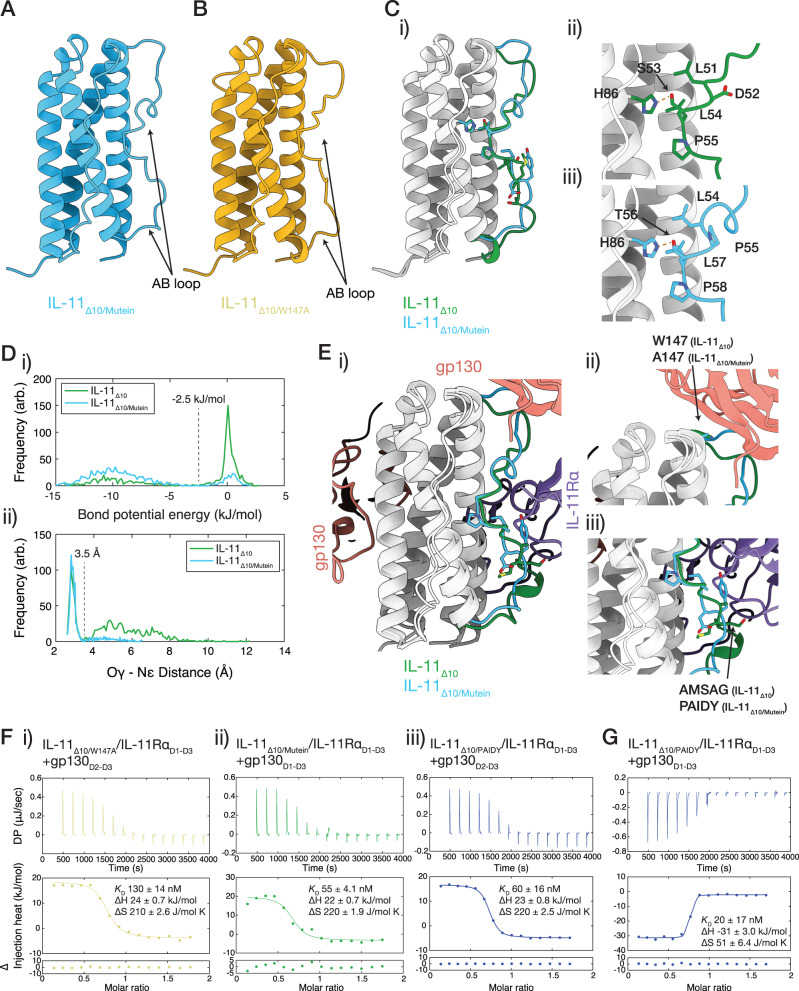
The structure of IL-11 Mutein, and mechanism of inhibition. **A** The structure of IL-11_Δ10_ Mutein. **B** The structure of IL-11_Δ10/W147A_. **C** Overlay of IL-11_Δ10_ Mutein and IL-11_Δ10_ (PDB ID: 6O4O^49^); (i) overall view of both structures, with the AB loop coloured as indicated in the figure. (ii) detail of the AB loop, showing changes in loop-core interaction that occur as a result of the PAIDY mutations in IL-11 Mutein. **D** MD analysis of the hydrogen bond between S53/T56 and H86. (i) distribution of the estimated bond potential energy for IL-11Δ10 and IL-11Δ10 Mutein through the simulation, an approximate cut-off for hydrogen bonding is indicated. (ii) distance distribution for the distance between the donor oxygen (S53/T56 Oγ) and the acceptor nitrogen (H86 Nε) through the simulation, an approximate cut-off for hydrogen bonding is indicated. **E** Overlay of the structure of IL-11_Δ10_ Mutein with the structure of the IL-11Rα_D1-D3_/gp130_D1-D3_/ IL-11_Δ10_ complex. (i) overall view. (ii) detail of the site-III interface, with the W147/A147 residue shown. (iii) detail of the AB loop, with the AMSAG/PAIDY residues shown. **F** Representative ITC data for (i) the interaction between the IL-11_Δ10/W147A_/IL-11Rα_D1-D3_ binary complex and gp130_D2-D3_; (ii) the interaction between the IL-11_Δ10/Mutein_/IL-11Rα_D1-D3_ binary complex and gp130_D1-D3_; and (iii) the interaction between the IL-11_Δ10/PAIDY_/IL-11Rα_D1-D3_ binary complex and gp130_D2-D3_ complex. Representative of *n* = 3 independent experiments. **G** Representative ITC data for the interaction between the IL-11_Δ10/PAIDY_/IL-11Rα_D1-D3_ binary complex and gp130_D1-D3_. Representative of *n* = 3 independent experiments. For complete thermodynamic parameters, see Supplementary Table 4.

We solved crystal structures of IL-11_Δ10/Mutein_ and IL-11_Δ10/W147A_ at resolutions of 1.8 Å and 1.5 Å, respectively, to understand the structural consequences of the mutations (Fig. 6A, B; Supplementary Fig. 11A–D; Supplementary Table 2). SAXS experiments show that the crystal structures are representative of the solution structure, and SV-AUC indicates that both proteins are monomeric in solution (Supplementary Fig. 11E, F; Supplementary Fig. 12).

The structure of IL-11_Δ10/W147A_ is similar to the structure of IL-11_Δ10_ (Supplementary Fig. 11Gi; RMSD 0.3 Å relative to IL-11_Δ10_^49^), indicating that the W147A mutation does not alter the structure. In contrast, the AB loop of IL-11_Δ10/Mutein_ shows significant conformational shifts relative to the WT structure caused by alterations in key contacts between the loop and the four-α-helical bundle (Fig. 6Ci; Supplementary Fig. 11Gii; RMSD 1.3 Å relative to IL-11_Δ10_^49^). The nature of the crystal contacts formed by the AB loop is similar in each crystal structure, comprising primarily contacts with the AB loop and CD loop of neighbouring molecules (Supplementary Fig. 11C, D), with the bulk of the loop surrounded by solvent. This suggests that the crystal packing does not constrain the configuration of the AB loop.

In IL-11_Δ10_^49^ and IL-11_Δ10/W147A_, a hydrogen bond is formed between the side chains of S53 within the AB loop and H86 on helix B (Fig. 6Cii). In contrast, in IL-11_Δ10/Mutein_ this hydrogen bond is formed between T56 of the loop and H86 (Fig. 6Ciii; for overlay see Supplementary Fig. 11H). Furthermore, key hydrophobic interactions between AB loop residues L51, L54 and P55 and the helical bundle of IL-11_Δ10_ and IL-11_Δ10/W147A_ (Fig. 6Cii; Supplementary Fig. 11Ii) are mediated by L54, L57 and P58 in IL-11_Δ10/Mutein_ (Fig. 6Ciii; Supplementary Fig. 11Iii). Thus, the ^58^PAIDY^62^ mutations alter the position of the AB-loop, shifting the register of key loop-core interactions by three residues, while maintaining the composition of the interacting residues. This register change is due to a shift of the LX(S/T)LP motif that mediates loop-core contacts: In IL-11_Δ10_ and IL-11_Δ10/W147A_ the motif is ^51^LDSLP^55^, while in IL-11_Δ10/Mutein_ the interacting motif is ^54^LPTLP^58^. The result is that the bulk of the loop is shifted toward site-III and the N-terminal turn of helix-B is unfolded. We note that this configuration of the C-terminal segment of the AB-loop and N-terminal turn of helix-B resembles the structure of wild-type IL-11_Δ10_ bound within the hexameric complex.

To assess the dynamics of the AB loop, we performed 1 μs molecular dynamics simulations of both IL-11_Δ10_ and IL-11_Δ10/Mutein_ (Fig. 6D; Supplementary Fig. 13; Supplementary Movies 4, 5). The overall dynamic profile of the four-α-helical core of the two proteins is very similar (Supplementary Fig. 13A–C). We analysed the stability of the IL-11_Δ10_ S53-H86 hydrogen bond and the IL-11_Δ10/Mutein_ T56-H86 hydrogen bond throughout the simulation by calculating the distance between the donor (S53 Oγ or T56 Oγ) and the acceptor (H86 Nε), and the distribution of the potential hydrogen bond energy (Fig. 6D; Supplementary Fig. 13D–F). Using a hydrogen bond energy cut-off of −2.5 kJ/mol, S53 of IL-11_Δ10_ is hydrogen bonded to H86 for 33% of the simulation, while T56 of IL-11_Δ10/Mutein_ is hydrogen bonded to H86 for 84% of the trajectory. This increased persistence of the hydrogen bond suggests that the AB loop conformation of IL-11_Δ10/Mutein_ is more stable than that of IL-11_Δ10_. Differential scanning fluorometry (DSF) analysis revealed that the temperature of hydrophobic exposure (*T*_h_^69^) of IL-11_Δ10/Mutein_ was 88.7 ± 0.16 °C indicating significantly higher thermal stability than both IL-11_Δ10_ (*T*_h_ 84.8 ± 0.39 °C, *p* = 0.003 vs IL-11_Δ10/Mutein_) and IL-11_Δ10/W147A_ (*T*_h_ 87.3 ± 0.29 °C, *p* = 0.04 vs IL-11_Δ10/Mutein_) (Supplementary Fig. 14A).

Together these data indicate that altered loop-core interactions brought about by the ^58^PAIDY^62^ mutations in IL-11_Δ10/Mutein_ result in a new, stabilised position of the AB loop. We propose that this improved stability is due to the backbone structural restraints introduced in the IL-11_Δ10/Mutein_ LX(S/T)LP motif by P55 at the X position.

### The AB loop of IL-11 Mutein acts in combination with the W147A mutation to block hexamer formation at site-III

Our IL-11 complex structures show that the N-terminal section of the AB loop contacts gp130 at site-III, suggesting that the altered conformation and stability of the AB loop in IL-11_Δ10/Mutein_ could alter interactions at this site. Superposition of the crystal structure of IL-11_Δ10/Mutein_ with an IL-11 molecule of our gp130_D1-D3_ complex structure (Fig. 6E) revealed that the N-terminal part of the AB-loop of IL-11_Δ10/Mutein_ clashes sterically with D1 of gp130 at site-III in the complex (Fig. 6Eii). This observation suggested that the altered conformation of the AB-loop acts to disrupt gp130 binding at site-III.

To investigate this possibility, we determined the affinities of the IL-11_Δ10/W147A_/IL-11Rα_D1-D3_, IL-11_Δ10/PAIDY_/IL-11Rα_D1-D3_, and IL-11_Δ10/Mutein_/IL-11Rα_D1-D3_ binary complexes for gp130 (Fig. 6F–G; Supplementary Table 4). The IL-11_Δ10/W147A_/IL-11Rα_D1-D3_ binary complex binds gp130_D2-D3_, forming a trimeric complex, with similar affinity to IL-11_Δ10_ (*K*_D_ 130 ± 14 nM, *p* = 0.15 vs IL-11_Δ10_; Fig. 6Fi). In contrast, the interaction of IL-11_Δ10/Mutein_/IL-11Rα_D1-D3_ with gp130_D1-D3_ and IL-11_Δ10/PAIDY_/IL-11Rα_D1-D3_ with gp130_D2-D3_ to form a trimer was significantly higher affinity than WT IL-11_Δ10_, with *K*_D_ of 55 ± 4 nM, (*p* = 0.01 vs IL-11_Δ10_) (Fig. 6Fii) and 60 ± 16 nM (*p* = 0.04 vs IL-11_Δ10_) (Fig. 6Fiii), respectively. Interestingly, IL-11_Δ10/PAIDY_/IL-11Rα_D1-D3_ binds gp130_D1-D3_ to form the hexameric complex with similar affinity compared to IL-11_Δ10_ (*K*_D_ 20 ± 17 nM, *p* = 0.2 vs IL-11_Δ10_; Fig. 6G; Supplementary Table 4).

These ITC results show that the PAIDY mutations increase site-II affinity but are insufficient to disrupt hexamer formation in the absence of the W147A mutation These observations are consistent with our biological assays (Fig. 4), indicating that the synergistic effects of the PAIDY and W147A mutations are required for the potent antagonist function of IL-11 Mutein.

The increase in site-II affinity of IL-11_Δ10/Mutein_ and IL-11_Δ10/PAIDY_ was surprising, given that the mutations in both proteins lie distant from the site-II interface (Fig. 6E). This increased affinity may be explained by the slower dissociation rate of the IL-11_Δ10/Mutein_/IL-11Rα_D1-D3_ complex relative to the IL-11_Δ10_/IL-11Rα_D1-D3_ complex. In this case, the increased residence time of IL-11_Δ10/Mutein_ bound to IL-11Rα_D1-D3_ increases the period that the composite site-II binding interface for gp130 is intact and, therefore, increases the frequency of productive binding collisions by gp130. Thus, the microscopic association rate of gp130 is increased with little effect on its dissociation rate. Alternatively, the altered site-II interaction may reflect modified dynamics of the bound cytokine variant or subtle adjustment of its binding pose on IL-11Rα induced by the ^58^PAIDY^62^ mutations.

Our ITC and SPR results, in combination with the structures of the IL-11 signalling complex, IL-11_Δ10/Mutein_, and IL-11_Δ10/W147A_ show that the ^58^PAIDY^62^ mutations in IL-11 Mutein significantly alter the interaction of the cytokine with both IL-11Rα and gp130 at all three binding sites, which likely underpins the efficacy of IL-11 Mutein as an IL-11 signalling inhibitor. The slower dissociation rate at site I and increased affinity at site II enhance the ability of IL-11_Δ10/Mutein_ to compete with native IL-11 for binding to IL-11Rα and gp130, thereby contributing to the competitive inhibition mechanism. These results also show that the AB loop in IL-11 is a critical region for the formation of the signalling complex, which will guide future design of inhibitors.

## Discussion

**Fig. 7:**
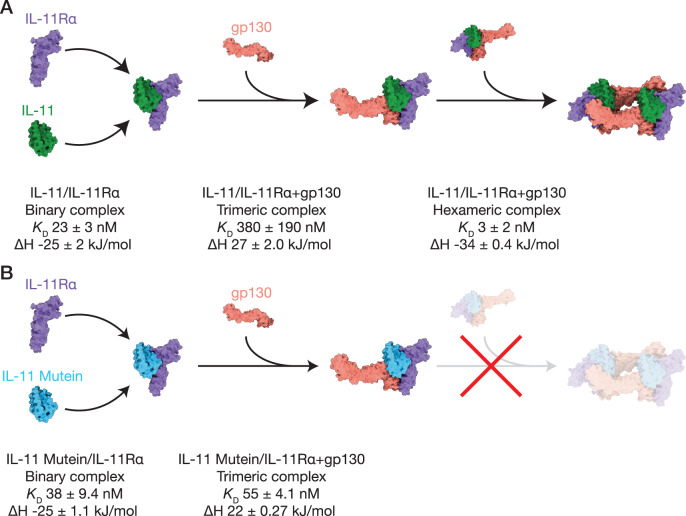
Schematic of IL-11 complex assembly, and mechanism of action of IL-11 Mutein. **A** Stoichiometry and thermodynamics of the three-step cooperative assembly of the IL-11 signalling complex. IL-11 and IL-11Rα form a binary complex that subsequently binds the first molecule of gp130 to form a trimeric intermediate. Two trimers then associate, resulting in a hexameric complex comprising two copies each of IL-11, IL-11Rα, and gp130. **B** The mechanism of IL-11 Mutein inhibition. IL-11 Mutein binds IL-11Rα and the first molecule of gp130 to form a trimeric complex. However, the IL-11 Mutein/IL-11Rα/gp130 trimer cannot dimerise to form a hexameric complex and, consequently, signalling is abolished. IL-11 Mutein competitively inhibits IL-11 signalling through a reduced rate of dissociation from IL-11Rα and increased affinity for the first molecule of gp130, relative to IL-11. Complexes and subunits are displayed as solvent-accessible molecular surfaces.

IL-11 signalling has been implicated in a number of human diseases and is pre-clinically validated as a therapeutic target, prompting significant investment in therapeutic development, with the first clinical trials imminent. However, informed development of IL-11 signalling inhibitors has been hindered by a lack of structural knowledge of the IL-11 complex, and a lack of understanding of the mechanism of action of existing IL-11 signalling inhibitors. We report the structures of the IL-11 signalling complex, including information on the position and dynamics of the extracellular domains of gp130, which have previously eluded high-resolution structural characterisation. We show that the complex forms in three steps (Fig. 7) involving significant conformational rearrangement of the cytokine that is coordinated by a key arginine residue, R169. These conformational changes of IL-11 upon complex formation are significantly larger than those observed for other IL-6 family members, IL-6 and LIF, and the nature of the interactions forming the IL-11 complex is distinct^35,37,70^.

The formation of the signalling complex results in activation of JAKs bound to the cytoplasmic region of gp130, mainly JAK1 and to a lesser extent JAK2 and TYK2. Activated JAKs subsequently phosphorylate tyrosine residues of gp130 resulting in activation of signalling pathways, including STAT, ERK, MAPK and phosphoinositide 3-kinase (PI3K) pathways that mediate biological outcomes^28,29^. Our data on the nature and dynamics of the membrane-proximal domains of gp130 have important implications for understanding the molecular mechanisms of JAK activation and propagation of downstream signalling pathways. The observation that D4-D6 of gp130 are flexible with respect to the membrane distal domains (D1-D3) provides information on the potential relative positions of the two D6 domains within the complex that may affect positioning of the transmembrane and cytoplasmic domains. This has implications for the relative positioning and activation of the bound JAK molecules and will inform further interrogation of signalling complexes including transmembrane and cytoplasmic machinery. In future, this knowledge may also inform how the different downstream signalling pathways are co-activated, or individually activated in disease, as evidence emerges that there may be a cell context dependent IL-11 mediated activation of STAT3 pro-survival pathways compared to IL-11 mediated ERK driven proliferation and apoptosis in disease^71^. Importantly, by defining the structural and biophysical dynamics of gp130 within the signalling complex our data enable a better understanding of the pathogenicity of emerging cytokine selective variants in IL6ST^64,72–77^, as well as variants in IL11R^78,79^ and IL11^80,81^, for which there are no targeted therapeutic opportunities for patients.

Our characterisation of the IL-11 signalling inhibitor, IL-11 Mutein, shows that it is highly effective at blocking signalling complex formation and potently inhibits signalling in a range of human cancer cell lines, highlighting the breadth of disease applications for emerging IL-11 signalling therapeutics. In contrast, the point mutant, IL-11 W147A, does not antagonise signalling by wild-type IL-11, contradicting previous reports^44,66,67^. In particular, our data show the importance of the IL-11 Mutein AB loop in modulating interactions with IL-11Rα and both gp130 molecules of the complex leading to inhibition of signalling (Fig. 7). Thus, engineering of the AB loop may be a general path for generation of antagonist variants of class I cytokines. Our results functionally and mechanistically validate IL-11 Mutein as a tool IL-11 signalling inhibitor and provide a straightforward, high-yield method for producing IL-11 Mutein suitable for laboratory studies of IL-11 biology.

Overall, the results presented here will facilitate significant advancement of IL-11 biology and will appreciably aid the development of therapeutic agents targeting IL-11 signalling and signalling by related cytokines.

## Methods

### Protein expression and purification

Throughout this work, we used an N-terminally truncated form of IL-11 (IL-11_Δ10_), and a C-terminally truncated form of IL-11Rα (IL-11Rα_D1-D3_), containing the structured extracellular domains characterised previously^49^. We used gp130 constructs containing the D1-D3 domains of gp130 (gp130_D1-D3_), D2-D3 domains (gp130_D2-D3_), or D1-D6 domains of gp130, which comprise the complete extracellular region of gp130 (gp130_EC_)^58^.

Human IL-11, IL-11 mutants, and IL-11Rα constructs were expressed and purified as previously described^49^. Human gp130 constructs were expressed using the same insect cell expression method as IL-11Rα^49^. Human IL-11 and IL-11 mutants, N-terminally fused to a His_6_ tag, maltose binding protein and a TEV protease cleavage site were expressed in BL21(DE3) *Escherichia coli* cells, and purified using sequential nickel-affinity, cation exchange and gel filtration chromatography. Cleavable tags were removed using TEV protease. IL-11Rα constructs and gp130 constructs, N-terminally fused to a honeybee-melittin signal peptide, His_8_ tag, and TEV cleavage site were expressed in *Spodoptera frugiperda* Sf21 (Invitrogen cat. 11497013) cells using baculovirus. Proteins were purified from the conditioned media using nickel-affinity chromatography, followed by tag removal using TEV protease and gel filtration chromatography.

For coupling to a streptavidin SPR chip, we generated IL-11_Δ10_ and IL-11_Δ10/Mutein_ constructs with a C-terminal avitag^68^, which was biotinylated in vitro in *Escherichia coli* BL21(DE3) cells. These fusion proteins were purified using identical methods to IL-11_Δ10_, and the degree of biotinylation was assessed using mass spectrometry.

### Purification of the IL-11 signalling complex

The IL-11 signalling complex was prepared by mixing equimolar amounts of IL-11_Δ10_, IL-11Rα_D1-D3_ and gp130_D1-D3_/gp130_EC_. The complex was incubated on ice for ~1 h, then applied to a Superdex 200 10/30 size exclusion column (Cytiva cat. 28990944), pre-equilibrated in TBS pH 8.0. Fractions containing the signalling complex were pooled and concentrated to 1-5 mg/mL. Purity of the complex was assessed using native-PAGE electrophoresis and sedimentation-velocity analytical ultracentrifugation. The IL-11_Δ10_/IL-11Rα_D1-D3_/gp130_EC_ complex was prepared using an identical method, although the complex used for cryo-EM was not size exclusion purified, which provided improved particle dispersion.

### Cryo-electron microscopy—data collection and 3D reconstruction

Cryo-EM was performed at the Bio21 Institute Ian Holmes Imaging Centre. The IL-11_Δ10_/IL-11Rα_D1-D3_/gp130_D1-D3_ complex (0.5 mg/mL) was blotted onto UltrAuFoil grids (R2/2, Quantifoil Micro Tools GmbH) that had been subjected to glow discharge (15 mA for 30 s). The sample was applied to the grid at 95% humidity, 4 °C, in a Vitrobot Mark IV (FEI) and blotted for 2 s with a blot force of −1, before plunging in liquid ethane. Grids were imaged using a Gatan K2 direct detector mounted on a Talos Arctica (FEI, Hillsborough, Oregon) with a 70-µm objective aperture. The detector was operated in super-resolution counting mode at 0.655 Å/ pixel (100,000 × magnification) with a defocus range of −0.8 to −2.0 µm. 40 frames per movie were acquired for a total dose of 50 electrons Å^-2^. Movies were acquired with specimen grids at tilts of 0° and 35° to increase particle orientation distribution.

The IL-11_Δ10_/IL-11Rα_D1-D3_/gp130_EC_ complex (1.5 mg/mL) in the presence of 1–2 mM *n*-dodecyl β-D-maltoside was blotted onto UltrAuFoil grids (R2/2, Quantifoil Micro Tools GmbH) that had been subjected to glow discharge (15 mA for 30 s). The sample was applied to the grid at 95% humidity, 4 °C, in a Vitrobot MarkIV (FEI) and blotted for 2 s with a blot force of −1, before plunging in liquid ethane. Grids were imaged using a Gatan K2 direct detector mounted on a Talos Arctica (FEI) with a 70-µm objective aperture. The detector was operated in counting mode. The complex was imaged at 1.31 Å/ pixel (100,000 × microscope magnification) with a defocus range of −0.8 to −2.0 µm. 40 frames per movie were acquired for a total dose of 50 electrons Å^−2^.

Processing of data for the IL-11_Δ10_/IL-11Rα_D1-D3_/gp130_D1-D3_ complex was carried out using RELION-3.0^82,83^ (Supplementary Fig. 1A). Movie motion was corrected using MotionCor2.1^84^. Cryosparc 2.1^63^ was used for CTF estimation using the patch CTF estimation routine. A total of 3,082,536 particles were extracted from 2,010 motion-corrected movies. After 2D class averaging in Cryosparc 2.1^63^, 625,866 particles were retained and were re-extracted in RELION-3.0 with the original defocus from Cryosparc 2.1^63^. After 3D classification 204,455 particles were used for the final 3D refinement. Final refinement with C2 symmetry yielded a map with a resolution of 3.5 Å. Resolution was estimated using gold standard FSC = 0.143 calculated using a relaxed solvent map. Maps were sharpened using *phenix.auto_sharpen*^85^. Local resolution maps were calculated using *Resmap*^86^. Buried surface area was determined using *PISA*.

Processing of data for the IL-11_Δ10_/IL-11Rα_D1-D3_/gp130_EC_ complex was carried out using Cryosparc 2.1^63^ (Supplementary Fig. 1B). Movie motion was corrected using MotionCor2.1^84^. Cryosparc 2.1^63^ was used for CTF estimation using the patch CTF estimation routine. WARP^87^ was used to pick 694,360 particles from 6,861 motion corrected movies. After 3 rounds of 2D classification, particles were submitted to heterogenous refinement and one further 2D classification leading to a final particle number of 125,373. These particles were used for standard refinement (4.0 Å) followed by a final non-uniform refinement using C2 symmetry leading to a map with a resolution of 3.8 Å.

### Cryo-electron microscopy—model building and refinement

For the IL-11_Δ10_/IL-11Rα_D1-D3_/gp130_D1-D3_ complex, published models of IL-11^49^ (PDB ID: 6O4O), gp130 D1-D3 (chain A from PDB ID: 1I1R^34^), and the IL-11Rα chain from an antibody-bound structure of IL-11Rα were docked into the cryo-EM density map of the IL-11_Δ10_/IL-11Rα_D1-D3_/gp130_D1-D3_ complex using *UCSF Chimera*^88^ to generate an initial model of the complex. This model was refined using *phenix.real_space_refine*^89^, followed by manual model-building in *Coot*^90^ and automated refinement in *phenix.real_space_refine*. Strict NCS was enforced in refinement. Reference model restraints were used throughout refinement, using the initial models as the reference models.

For the IL-11_Δ10_/IL-11Rα_D1-D3_/gp130_EC_ complex, published models of IL-11^49^ (PDB ID: 6O4O), gp130 D1-D3 (chain A from PDB ID: 1I1R^34^) gp130_D4-D6_^58^ (PDB ID: 3L5I), and the IL-11Rα chain from an antibody-bound structure of IL-11Rα were docked into the cryo-EM density map of the IL-11_Δ10_/IL-11Rα_D1-D3_/gp130_D1-D3_ complex using *UCSF Chimera*^88^ to generate an initial model of the complex. Refinement was conducted in the same manner as the IL-11_Δ10_/IL-11Rα_D1-D3_/gp130_D1-D3_ complex. The deposited model of the IL-11_Δ10_/IL-11Rα_D1-D3_/gp130_EC_ complex did not include the D5-D6 domains of gp130; we prepared a second model, which we did not deposit to the PDB, which included the D5-D6 domains of gp130. This model is included in SASBDB deposition SASDLN3. The structure of IL-11Rα used as the initial model for cryo-EM model building is not currently available due to intellectual property considerations. The same analysis of the cryo-EM data can be repeated with the available structure 6O4P^49^.

Geometry validation was performed using the *phenix.validation_cryoem* tool (incorporating *MOLProbity*) and *EMRinger*^91,92^. Structures were visualised using *UCSF Chimera*^88^. Structures were aligned using the *Matchmaker* algorithm in *UCSF Chimera*^88^ or the *CE* algorithm^93^ in *PyMOL* 2.2. Map-model FSC curves were calculated in *Phenix*. Figures were prepared using *UCSF Chimera*^88^. The conclusions regarding the binding interactions described are supported by the gp130_D1-D3_ and gp130_EC_ complex structures, and the crystal structure of the complex. Figure 2 was prepared with the gp130_D1-D3_ complex structure, as this structure is supported by the highest resolution data.

For the IL-11_Δ10_/IL-11Rα_D1-D3_/gp130_EC_ complex 3D variability analysis with 3 modes to solve was performed in C1 symmetry on the final 125,373 particles used for refinement without symmetry expansion. 3D variability display was used in intermediate mode with 9 frames for each mode solved.

### Protein crystallisation and X-ray data collection

The IL-11 complex for crystallisation was prepared by combining equimolar amounts of IL-11_FL_, IL-11Rα_EC_ and gp130_D1-D3_, followed by purification of the complex using gel filtration. Crystals of the IL-11 signalling complex were grown at 20 °C in 180 mM magnesium chloride, 15.3% PEG 3350, 100 mM potassium sodium tartrate, 90 mM sodium HEPES pH 7.25 and 1.8% tert-butanol. Crystallisation drops were prepared by mixing equal volumes of the precipitant and IL-11 signalling complex (at 3 mg/mL), and 10 μL of a seed stock (prepared according to the method of Luft and DeTitta)^94^. Crystals were flash-cooled in liquid nitrogen directly from crystallisation drops, and X-ray diffraction data were collected at 100 K at the Australian Synchrotron MX2 beamline^95^. X-ray data collection statistics are tabulated in Supplementary Table 2.

Initial crystals of IL-11_Δ10/Mutein_ were obtained using a similar screening approach to IL-11_Δ10_^49^, in the precipitant 30% PEG 3350, 0.2 M ammonium sulfate, 0.1 M Tris pH 8.5, 20 °C. These initial crystals were used to prepare a microseed stock^94^. Large, single plates of IL-11_Δ10/Mutein_ grew in the condition 27% PEG 3350, 0.1 M bis-tris propane pH 9, 0.2 M ammonium sulfate, 5 mM praseodymium chloride, 20 °C. Crystallisation drops were produced by mixing 1.5 μL precipitant, 1.5 μL IL-11_Δ10/Mutein_ (5 mg/mL) and 0.5 μL seed. Crystals of IL-11_Δ10/Mutein_ were large plates, with approximate dimensions 200 × 200 × 5 μm. A similar approach was used to crystallise IL-11_Δ10/W147A_. These mutants were cross seeded with seed generated from IL-11_Δ10_ or IL-11_Δ10/Mutein_ crystals and grew in very similar conditions to IL-11_Δ10/Mutein_. Crystals of IL-11_Δ10/W147A_ were rods, similar to crystals of IL-11_Δ10_^49^.

Crystals were flash-cooled in liquid nitrogen directly from crystallisation drops, and X-ray diffraction data were collected at 100 K at the Australian Synchrotron MX2 beamline^95^. X-ray data collection statistics are tabulated in Supplementary Table 2.

### X-ray data processing and structure refinement

For the IL-11 signalling complex, diffraction data were indexed, integrated and scaled using *XDS*^96^, analysed using *POINTLESS*^97^ and merged using *AIMLESS*^98^ from the *CCP4* suite. Due to the highly anisotropic nature of the data, an ellipsoidal resolution cutoff was applied using the *STARANISO*^99^ server. Initial phase estimates were obtained using molecular replacement with *Phaser*^100^ using the cryo-EM structure of the IL-11_Δ10_/IL-11Rα_D1-D3_/gp130_D1-D3_ complex as the search model. Refinement was performed in *phenix.refine*^101^, including several rounds of simulated annealing early in the refinement process. Strict NCS restraints (NCS constraints), reference model restraints using the high-resolution structures of the complex components, and Ramachandran restraints were employed. Iterative model-building was performed in *Coot*^90^ using NCS-averaged maps. NCS map averaging was performed in *Coot*. N-linked glycans were defined in the electron density and were included in the model. Refinement statistics are tabulated in Supplementary Table 2.

For IL-11_Δ10/Mutein_, and IL-11_Δ10/W147A_, diffraction data were indexed, integrated and scaled using *XDS*^96^, analysed using *POINTLESS*^97^ and merged using *AIMLESS*^98^, initial phase estimates were obtained using molecular replacement with *Phaser*^100^, using either our original structure of IL-11 (PDB ID: 4MHL)^81^ for IL-11_Δ10/Mutein_ or our high-resolution structure of IL-11_Δ10_ for IL-11_Δ10/W147A_ (PDB ID: 6O4O)^49^ as the search model. Auto-building with simulated annealing was performed in *phenix.autobuild* to reduce phase bias from the search model. Refinement was performed in *phenix.refine*^101^ with iterative manual building using *Coot*^90^. TLS refinement was performed using a single TLS group containing all protein atoms. Explicit riding hydrogens were used throughout refinement and included in the final model, the atomic position and *B* factors for hydrogens were not refined. Residues of all structures are numbered in an identical manner to our structure of IL-11_Δ10_ (PDB ID: 6O4O)^49^, reflecting the mature protein sequence after cleavage of signal peptide. Structures were aligned using the CE^93^ algorithm in *PyMOL* 2.2. Refinement statistics are tabulated in Supplementary Table 2.

### Analytical ultracentrifugation

SV-AUC experiments were conducted using a Beckman Coulter XL-I analytical ultracentrifuge or a Beckman Optima analytical ultracentrifuge, both equipped with UV-visible scanning optics. Samples were loaded into double-sector cells with quartz windows, and centrifuged using an An-60 Ti or An-50 Ti rotor at 50,000 rpm (209,625 *g*) and at 20 °C. Radial absorbance data was collected in continuous mode at 230, 250 or 280 nm. Sedimentation data were fit to a continuous sedimentation coefficient c(*s*) model, with floating frictional ratios using *SEDFIT*^102^. Buffer density, viscosity and the partial specific volume of the protein samples were calculated using *SEDNTERP* ^103^. For complexes, the partial specific volume used was 0.73 mL/g.

SE-AUC experiments were conducted using a Beckman Coulter XL-I analytical ultracentrifuge, equipped with UV-visible scanning optics. 160 μL of sample was loaded into double-sector cells and centrifuged using an An-60 TI rotor. To calculate M* for each component of the IL-11 signalling complex, proteins were diluted such that A_280_ ~ 0.35, and then centrifuged sequentially at 10,500 rpm (9,244 *g*), 17,000 rpm (24,232 *g*) and 28,000 rpm (65,738 *g*) until equilibrium was reached. For gp130_EC_, the three speeds were 8000 rpm (5,366 *g*), 10,000 rpm (8,385 *g*) and 16,000 rpm (21,465 *g*). For the IL-11_Δ10_/gp130_D1-D3_/IL-11Rα_D1-D3_ complex the complex was diluted such at A_250_ ~ 0.35 and then centrifuged sequentially at 5,300 rpm (2,355 *g*), 6,300 rpm (3,328 *g*) and 9,000 rpm (6,791 *g*). For the IL-11_Δ10_/gp130_EC_/IL-11Rα_D1-D3_ complex, the three speeds used were 4000 rpm (1,341 *g*), 5000 rpm (2,096 *g*) and 8000 rpm. (5,366 *g*) For all samples, M* was determined using a single-species analysis model in *SEDPHAT* ^104^. For the complexes, the sample was size exclusion purified prior to analysis, and sample purity was confirmed with SV-AUC prior to the SE-AUC experiment.

### Multi-angle light scattering

SEC-MALS data were collected using a Shimadzu LC-20AD HPLC, coupled to a Shimadzu SPD-20A UV detector, Wyatt Dawn MALS detector and Wyatt Optilab refractive index detector. Data were collected following in-line fractionation with a Zenix-C SEC-300 4.6 × 300 mm SEC column (Sepax Technologies, cat. 233300-4630), pre-equilibrated in 20 mM Tris, 150 mM sodium chloride pH 8.5, running at a flow rate of 0.35 mL/min. 10 µL of sample was applied to the column at a concentration of ~2 mg/mL. MALS data were analysed using ASTRA v.7.3.2.19 (Wyatt). The MALS detector response was normalised using monomeric bovine serum albumin (BSA) (Pierce, cat. 23209). Protein concentration was determined using differential refractive index, using a dn/dc of 0.184.

### Small-angle X-ray scattering

SAXS experiments were conducted at the Australian Synchrotron SAXS/WAXS beamline^105–107^. The X-ray beam energy was 11,500 eV (*λ* = 1.078 Å), the sample-to-detector distance is noted in Supplementary Table 3. Data were collected following fractionation with an in-line size-exclusion chromatography column (Superdex 200 5/150 Increase, GE Healthcare, cat. 28990945) pre-equilibrated in TBS pH 8.5, 0.2% sodium azide. Data were reduced and analysed using Scatterbrain, CHROMIXS^108^ and ATSAS^108,109^, data were analysed using CRYSOL and DAMMIF/DAMAVER/DAMMIN^110–112^. No constant subtraction was applied in CRYSOL. A summary is given in Supplementary Table 3.

Models were prepared of the IL-11_Δ10_/IL-11Rα_D1-D3_/gp130_D1-D3_ complex, the IL-11_Δ10_/IL-11Rα_D1-D3_/gp130_D2-D3_ complex, the IL-11_Δ10_/IL-11Rα_D1-D3_/gp130_EC_ complex, and the IL-11_Δ10/Mutein_/IL-11Rα_D1-D3_/gp130_D1-D3_ complex based on the IL-11_Δ10_/IL-11Rα_D1-D3_/gp130_EC_ complex (see Supplementary Table 3 for the regions of the complex used) for fitting to scattering data in *CRYSOL*. To fit the IL-11_Δ10_/IL-11Rα_D1-D3_/gp130_EC_ complex data, the model of the gp130_EC_ complex with the D5-D6 domains included was used. For the crystal structure of the IL-11 signalling complex, one hexamer from the crystal structure was used. For IL-11_Δ10/Mutein_ and IL-11_Δ10/W147A_, the unmodified crystal structure coordinates were used.

Multi-state models of the gp130_EC_ complex were generated and fit to the gp130_EC_ complex SAXS data using the Multi-FoXS server^113^ (version main.d5bb161). Flexible residues were defined in the IL-11Rα D1-D2 linker, and the gp130_EC_ D3-D4, D4-D5 and D5-D6 linkers (Supplementary Table 3), the overall complex assembly was defined as a single rigid body, and 10,000 conformations were generated. The gp130_EC_ complex coordinates with D5-D6 domains included was fit to the SAXS data using the FoXS server^114^ for direct comparison.

### Isothermal titration calorimetry

Protein samples were buffer exchanged into TBS pH 8.5 using size exclusion prior to analysis by ITC. ITC data were collected using a MicroCal iTC 200 (GE Healthcare). Titrations were performed using 15 2.5 μL injections of the cytokine ligand, after an initial injection of 0.8 μL. IL-11Rα_D1-D3_ was present at a concentration of 10 μM and the concentration of IL-11 or the relevant IL-11 mutant in the syringe was 10-fold greater than the concentration of IL-11Rα. Following the formation of the cytokine/receptor complex, gp130_D1-D3_ or gp130_D2-D3_ was loaded in the syringe at a concentration 10-fold greater than the concentration of IL-11Rα in the cell, a subsequent titration of gp130 against the cytokine/IL-11Rα_D1-D3_ complex was then performed at 288 K. Titration data were integrated using *NITPIC*^115,116^, and analysed in *SEDPHAT*^117^ using a 1:1 interaction model as defined by the AUC data. Each titration was conducted in triplicate, values stated are the mean ± standard error of the mean. Significance of differences were calculated using a one-tailed paired *t*-test.

### Cell stimulations and phospho-Stat3 flow cytometry assay

Ba/F3 cells stably expressing human gp130 and IL-11Rα (generated at WEHI)^65^ were cultured in RPMI-1640 medium containing 10% v/v heat inactivated FBS, 1% v/v Penicillin-Streptomycin and 10 ng/mL recombinant IL-11_Δ10_ at 37 °C and 5% CO_2_. Cells were routinely tested for mycoplasma and confirmed to be negative prior to experiments.

Cancer cell lines MDA-MD-231, A549, U87-MG, BxPC3, and LnCap (Cell Bank Australia codes: 92020424, 86012804, 89081402, JCRB1448, 89110211, respectively) were cultured in RPMI-1640 medium containing 10% v/v heat inactivated FBS, 1% v/v Penicillin-Streptomycin, at 37 °C and 5% CO_2_, except for MDA-MD-231, which were cultured in DMEM. Each cell line was routinely tested for mycoplasma and confirmed to be negative prior to experiments.

Cells were seeded at 50,000 cells per well of a 96-well plate overnight and serum starved for 2 h prior to stimulation. To determine EC_50_, the cells were stimulated with the indicated concentration of IL-11_Δ10_, IL-11_Δ10/Mutein_, IL-11_Δ10/W147A_, or IL-11_Δ10/PAIDY_ prepared in Fixable Viability Dye (1/1000, eBioscience Fixable Viability Dye eFluor 506, Thermo Fisher Scientific cat. 65-0866-14) for 15 min at 37 °C. To determine IC_50_, the cells were incubated with the indicated concentration of 11_Δ10/Mutein_, IL-11_Δ10/W147A_, or IL-11_Δ10/PAIDY_ for 15 min at 37 °C followed by stimulation with 20 ng/mL IL-11_Δ10_ prepared in Fixable Viability Dye and incubated for a further 15 min at 37 °C.

Cells were harvested by centrifugation at 1600 rpm for 3 min and fixed in Cytofix Fixation Buffer (BD Biosciences, cat. 554655) for 10 min at 37 °C. Cells were then centrifuged and washed with Stain Buffer (BD Biosciences cat. 554656) and permeabilised with Phosphoflow Perm buffer III (BD Biosciences cat. 558050) for 30 min at 40 °C. Cells were then centrifuged, washed with Stain Buffer and stained with Phospho-STAT3 antibody (1/10, clone 4/P-STAT3, BD Biosciences cat. 557815) or Mouse IgG2a, κ Isotype control (1/10, clone MOPC-173 (RUO), BD Biosciences cat. 558053). Data were acquired on a BD Biosciences Fortessa instrument and analysed using FloJo 10.8.2 software. See Supplementary Fig. 9D for gating strategy. EC_50_/IC_50_ experiments were conducted in triplicate and values are presented as mean ± standard error of the mean. Statistical analysis was performed in GraphPad Prism 9.4.1.

### Molecular dynamics

All MD simulations were performed using NAMD 2.1.3b1^118^ and the CHARMM22 forcefield^118,119^ at 310 K in a water box with periodic boundary conditions. A model of each of IL-11_Δ10_ and IL-11_Δ10/Mutein_ was built based on the crystal structures, for residues with multiple orientations, only one was selected. The structures were solvated (box size 53.6 × 53.1 × 74.9 Å), and ions added to approximate final concentration of 0.15 M NaCl. The MD simulations were performed using 10 ps minimisation time, followed by 1050 ns MD.

Visualisation and analysis was performed in VMD 1.9.3^120^. A script was used in VMD to measure the distance and calculate the distance distribution between T56/S53 Oγ and H86 Nε throughout the simulation. A script was used to calculate the hydrogen bond potential energy and potential energy distribution of this interaction using a simple electrostatic model, based on the model used in the secondary structure analysis programme *DSSP* ^121^. Per-residue Cα RMSD and order parameter (*S*^2^) values were calculated using scripts in VMD (available at 10.5281/zenodo.8302141).

### Differential scanning fluorometry

Protein samples were analysed by DSF at a concentration of 0.1 mg/mL in TBS pH 8 + 0.02% sodium azide, with 2.5 × SYPRO Orange dye (Sigma Aldrich, cat. S5692). 20 μL of the sample was loaded into 96-well qPCR plate (Applied Biosystems), and four technical replicates of each sample were analysed. The plates were sealed, and samples heated in an Applied Biosystems StepOne Plus qPCR instrument, from 20 °C to 95 °C, with a 1% gradient. Unfolding data were analysed using a custom script in MATLAB r2019a (available at 10.5281/zenodo.8302206). The temperature of hydrophobic exposure (*T*_h_), was defined as the minimum point of the first derivative curve, and used to compare the thermal stability of different proteins^69^. All experiments were repeated three times, values are given as mean ± standard error of the mean. Significance of differences were calculated using a two-tailed paired *t*-test.

### Surface plasmon resonance

SPR experiments were conducted using a Biacore T200, at 25 °C, in TBS pH 8.5 + 0.05% Tween, running at 30 μL/min. Experimental conditions and buffer composition were chosen to optimise stability of the interacting partners while minimising non-specific interactions. Biotinylated IL-11_Δ10_-avi and IL-11_Δ10/Mutein_-avi were loaded onto separate channels on a SAHC 1500 M streptavidin chip (Xantec). Both proteins were immobilised until *R*_max_ was ~70. The chip was washed extensively with 1 M NaCl until the baseline was stable. A nine-point, two-fold dilution series of IL-11Rα_D1-D3_ was prepared, starting at a concentration of 500 nM. Each IL-11Rα dilution was injected over both flow cells in triplicate and reference-subtracted data was generated by subtracting the response from channels in which no protein was loaded from channels containing protein. Data were fit to a 1:1 kinetic model and the Biacore analysis software was used to determine association (*k*_a_) and dissociation (*k*_d_) rates, and the dissociation constant, *K*_D_. Dilution series were prepared and analysed in duplicate, with each complete replicate dilution series analysed separately. Values are given as mean ± standard error of the mean.

### Reporting summary

Further information on research design is available in the Nature Portfolio Reporting Summary linked to this article.

### Supplementary information

Supplementary Information

Description of Additional Supplementary Files

Supplementary Movie 1

Supplementary Movie 2

Supplementary Movie 3

Supplementary Movie 4

Supplementary Movie 5

Reporting Summary

### Source data

Source Data

## Data Availability

Cryo-EM maps generated in this study have been deposited in the Electron Microscopy Data Bank (EMDB) under accession codes EMD-27641 (IL-11_Δ10_/IL-11Rα_D1-D3_/gp130_D1-D3_ complex), EMD-27632 (IL-11_Δ10_/IL-11Rα_D1-D3_/gp130_EC_ complex). Atomic model coordinates generated in this study have been deposited in the Protein Data Bank (PDB) under accession codes 8DPS (IL-11_Δ10_/IL-11Rα_D1-D3_/gp130_D1-D3_ complex), 8DPT (IL-11_Δ10_/IL-11Rα_D1-D3_/gp130_EC_ complex). Structure factors and atomic model coordinates generated in this study have been deposited in the PDB under accession codes 8DPU (IL-11_Δ10_/IL-11Rα_D1-D3_/gp130_D1-D3_ complex), 8DPV (IL-11_Δ10/W147A_), 8DPW (IL-11_Δ10/Mutein_). Previously published coordinates used in this study are available in the PDB under accession codes 6O4O (IL-11_Δ10_), 1I1R (vIL-6 signalling complex), 3L5I (gp130_D4-D6_), 4MHL (IL-11), 1P9M (IL-6 signalling complex) and 1PVH (LIF/gp130 complex). The structure of IL-11Rα used as the initial model for cryo-EM model building is not currently available due to intellectual property considerations. The same analysis of the cryo-EM data can be repeated with the available structure 6O4P. SAXS data generated in this study and associated atomic model coordinates have been deposited in the Small Angle Scattering Biological Data Bank (SASBDB) with the following accession codes: SASDLM3 (IL-11_Δ10_/IL-11Rα_D1-D3_/gp130_D1-D3_ complex), (SASDLN3) (IL-11_Δ10_/IL-11Rα_D1-D3_/gp130_EC_ complex), SASDLP3 (IL-11_Δ10_/IL-11Rα_D1-D3_/gp130_D2-D3_ complex), SASDLS3 (IL-11_Δ10/Mutein_/IL-11Rα_D1-D3_/gp130_D1-D3_ complex), SASDLR3 (IL-11_Δ10/Mutein_), SASDLQ3 IL-11_Δ10/W147A_. Molecular dynamics protein structure files (PSF) and trajectories have been deposited on Figshare at 10.6084/m9.figshare.24043518 (IL-11_Δ10_ microsecond MD trajectory) and 10.6084/m9.figshare.24043527 (IL-11_Δ10/Mutein_ microsecond MD trajectory). Source data for the ITC data presented in Figs. 2, 5 and 6 was deposited on Figshare at 10.6084/m9.figshare.24293080. Source data for the SPR data presented in Fig. 5D was deposited on Figshare at 10.6084/m9.figshare.24293563. Other data are contained within the manuscript and Supplementary Information. Materials generated in this study may be obtained from the corresponding author for non-commercial research use via a materials transfer agreement. Source data are provided with this paper.

## Code availability

VMD scripts used for analysis of the MD trajectories are available on Zendo at 10.5281/zenodo.8302141. The MATLAB script used for DSF data analysis is available on Zendo at 10.5281/zenodo.8302206.
