# Supplementary material for: Structures of the interleukin 11 signalling complex reveal gp130 dynamics and the inhibitory mechanism of a cytokine variant
Source: Nat Commun. 2023 Nov 20;14:7543. doi: 10.1038/s41467-023-42754-w (PMC10662374; doi:10.1038/s41467-023-42754-w)
Supplement: Supplementary file 1 — Supplementary Information [file 41467_2023_42754_MOESM1_ESM.pdf]

Supplementary Information

Structures of the interleukin 11 signalling complex reveal gp130 dynamics and the inhibitory mechanism of a cytokine variant

Riley D. Metcalfe[1†*], Eric Hanssen[1,2,3*], Ka Yee Fung[4,5], Kaheina Aizel[1,4,5], Clara C. Kosasih[1,4,5], Courtney O. Zlatic[1], Larissa Doughty[1], Craig J. Morton[1^], Andrew P. Leis[2,4,5], Michael W. Parker[1,3,6], Paul R. Gooley[1], Tracy L. Putoczki[4,5‡], Michael D.W. Griffin[1,3,‡,#].

[1] Department of Biochemistry and Pharmacology, Bio21 Molecular Science and Biotechnology Institute, University of Melbourne, Victoria 3010, Australia
[2] Ian Holmes Imaging Centre, Bio21 Molecular Science and Biotechnology Institute, University of Melbourne, Victoria 3010, Australia
[3] ARC Centre for Cryo-electron Microscopy of Membrane Proteins, Bio21 Molecular Science and Biotechnology Institute, University of Melbourne, Parkville, Victoria, Australia
[4] Walter and Eliza Hall Institute of Medical Research, Parkville, Victoria 3052, Australia
[5] Department of Medical Biology, University of Melbourne, Parkville, Victoria, 3010, Australia
[6] St Vincent's Institute of Medical Research, Fitzroy, Victoria, 3065, Australia

[*]These authors contributed equally
[‡]These authors jointly supervised this work
[#] Corresponding author; e-mail: mgriffin@unimelb.edu.au
[†] Current Address: Center for Structural Biology, Center for Cancer Research, National Cancer Institute, Frederick, Maryland, USA
[^] Current Address: CSIRO Biomedical Manufacturing Program, Victoria 3168, Australia

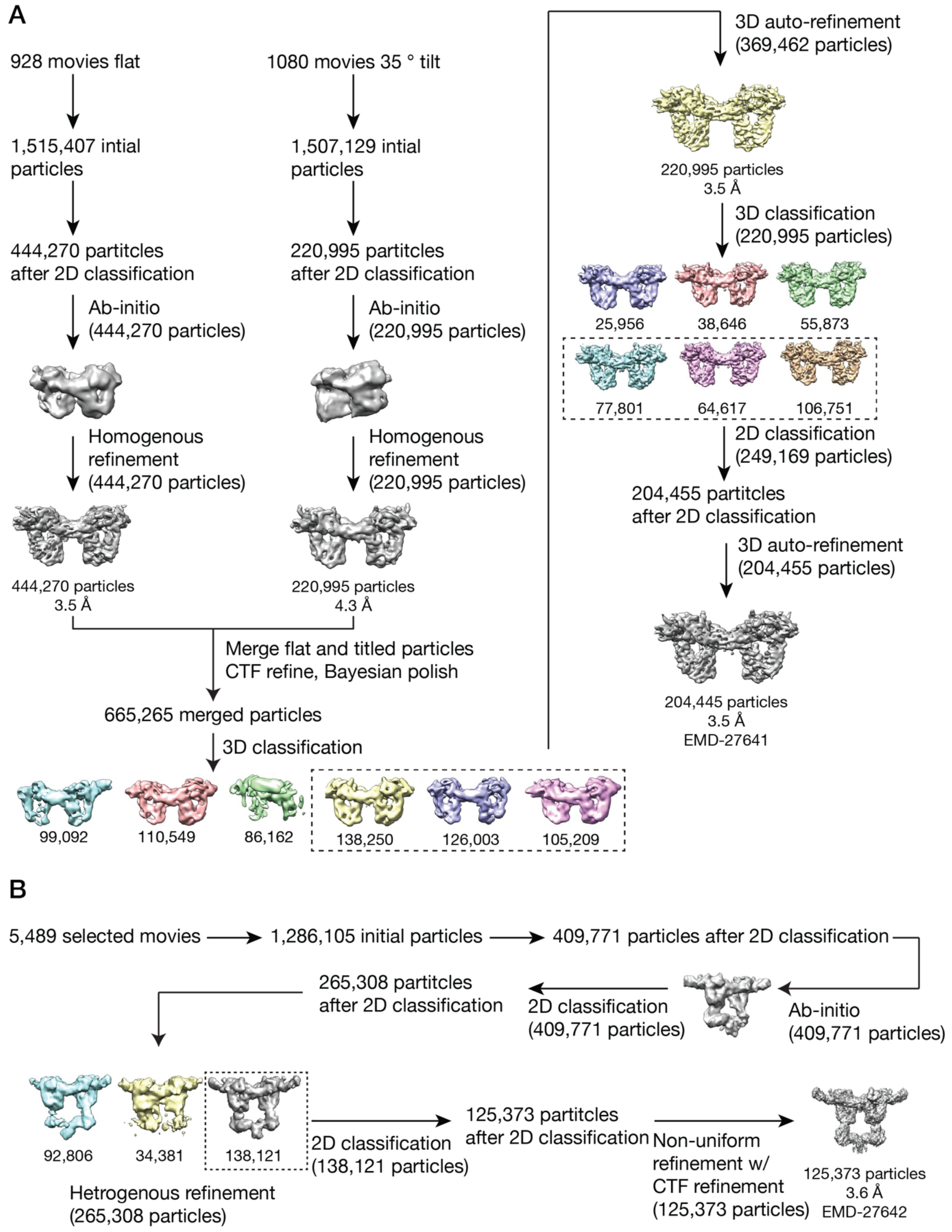

Supplementary Figure 1: Flow chart showing cryo-EM data processing steps for, A) the hexameric IL-11/IL-11Rα$_{D1\text{-}D3}$/gp130$_{D1\text{-}D3}$ complex and B) the hexameric IL-11/IL-11Rα$_{D1\text{-}D3}$/gp130$_{EC}$ complex.

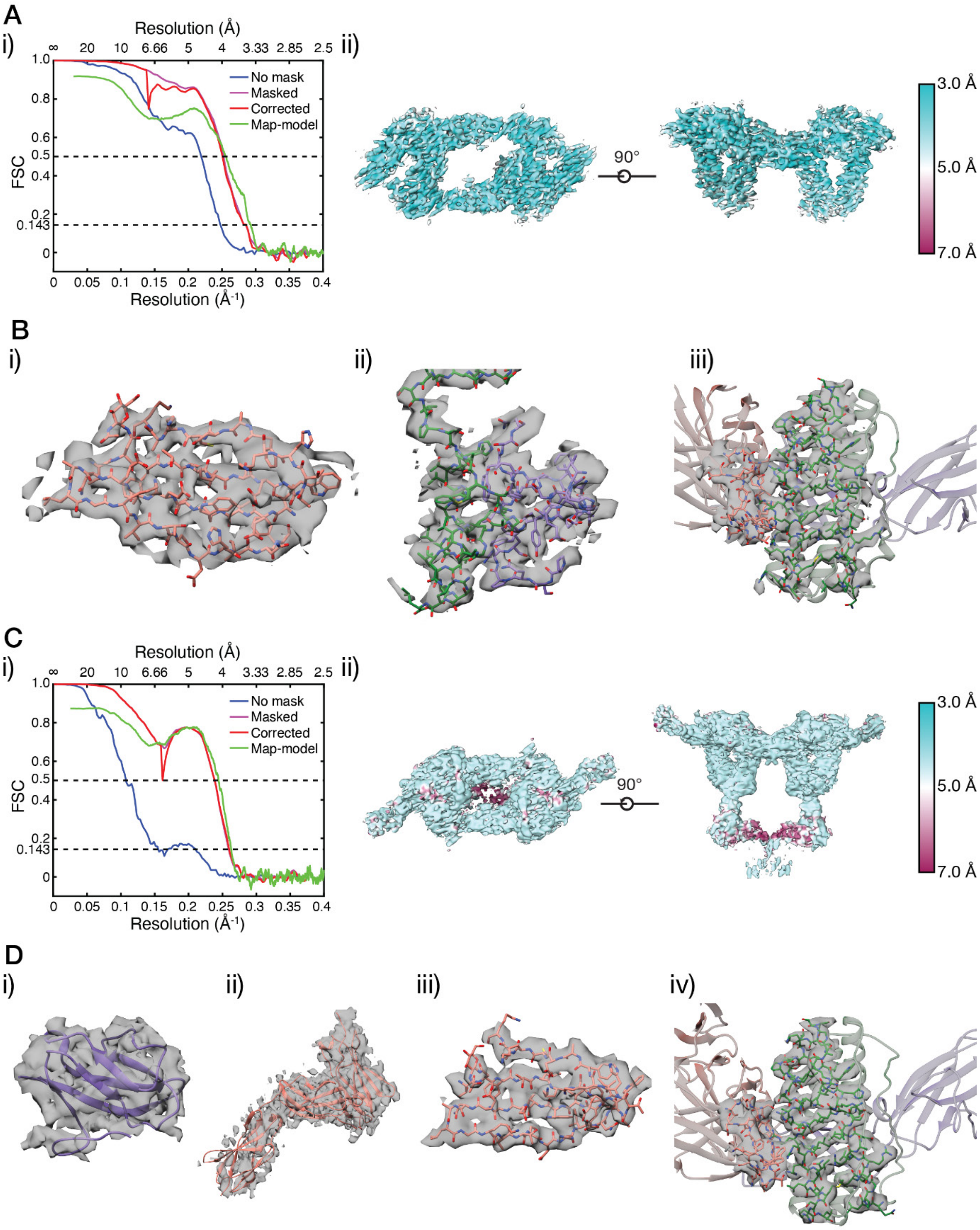

Supplementary Figure 2: Resolution estimation and representative cryo-EM density. A) Resolution estimation for the gp130$_{D1\text{-}D3}$ complex. i) Gold-standard half-map Fourier shell correlation (FSC) curves for unmasked (blue), masked (purple), and noise corrected masked maps (red), calculated in *Cryosparc*[1]. Map-model FSC, calculated in *Phenix*[2, 3], is shown in green. ii) Local resolution maps, calculated using *Resmap*[4]. B) Representative cryo-EM density contoured at 7 σ for the gp130$_{D1\text{-}D3}$ complex, coloured as in Figure 1. i) one of the β-sheets in gp130 D2; ii) the site-III interface; iii) the site-II interface. C) Resolution estimation for the gp130$_{EC}$ complex; i) Gold-standard half-map Fourier shell correlation (FSC) curves for

unmasked (blue), masked (purple), and noise corrected masked maps (red), calculated in *Cryosparc*[1]. Map-model FSC, calculated in *Phenix*[2, 3], is shown in green. ii) Local resolution maps, calculated using *Cryosparc*[1, 4]. D) Representative cryo-EM density for the $gp130_{EC}$ complex; i) IL-11Rα D1 contoured at 3 σ; ii) gp130 D4-D6 contoured at 3 σ; iii) one of the β-sheets in gp130 D2 contoured at 7 σ; iv) the site-III interface contoured at 7 σ.

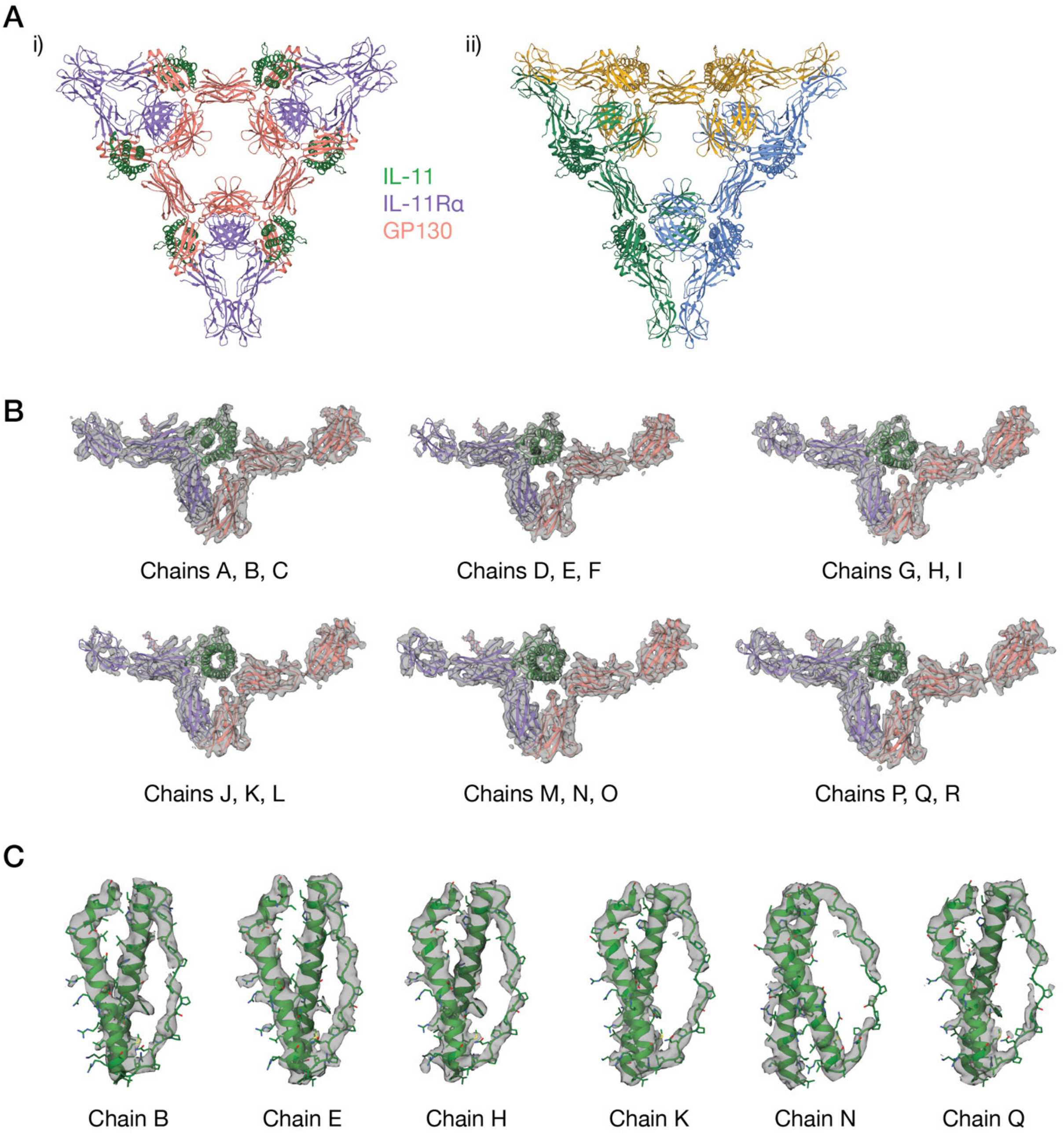

Supplementary Figure 3: Asymmetric unit and representative electron density for the IL-11 complex crystal structure. A) The asymmetric unit of the crystal structure of the IL-11 signalling complex, with three hexamers in the asymmetric unit, coloured according to molecule in i), each hexamer coloured differently in ii). B) Representative electron density for each trimer in the crystal structure. C) Representative electron density for the C and D helices in IL-11, from each trimer in the crystal structure. Density contoured at 1 σ, with missing F_{obs} not filled.

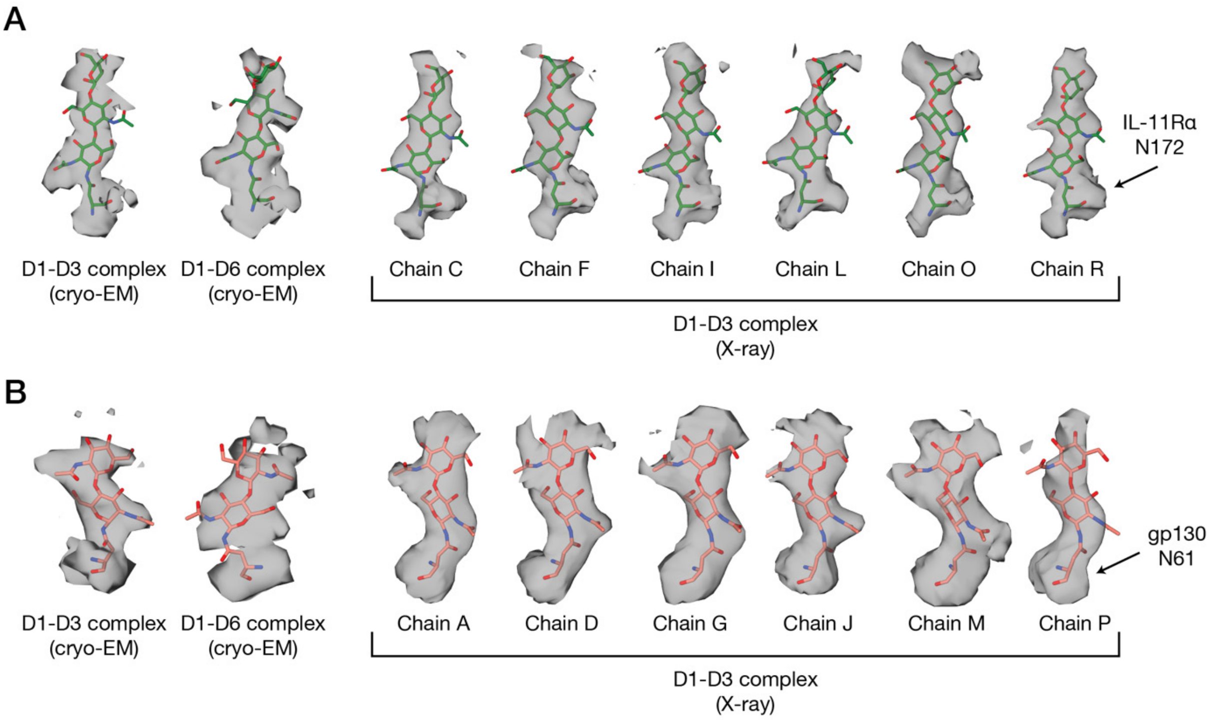

Supplementary Figure 4: Density supporting the position of glycans in the cryo-EM maps or X-ray electron density map. A) Density supporting the position of the glycan linked to N172 (indicated) in D2 of IL-11Rα. B) Density supporting the position of the glycan linked to N61 (indicated) in D1 of gp130. Electron density is contoured at 1 σ, with missing F_{obs} not filled and cryo-EM density is contoured at 7 σ.

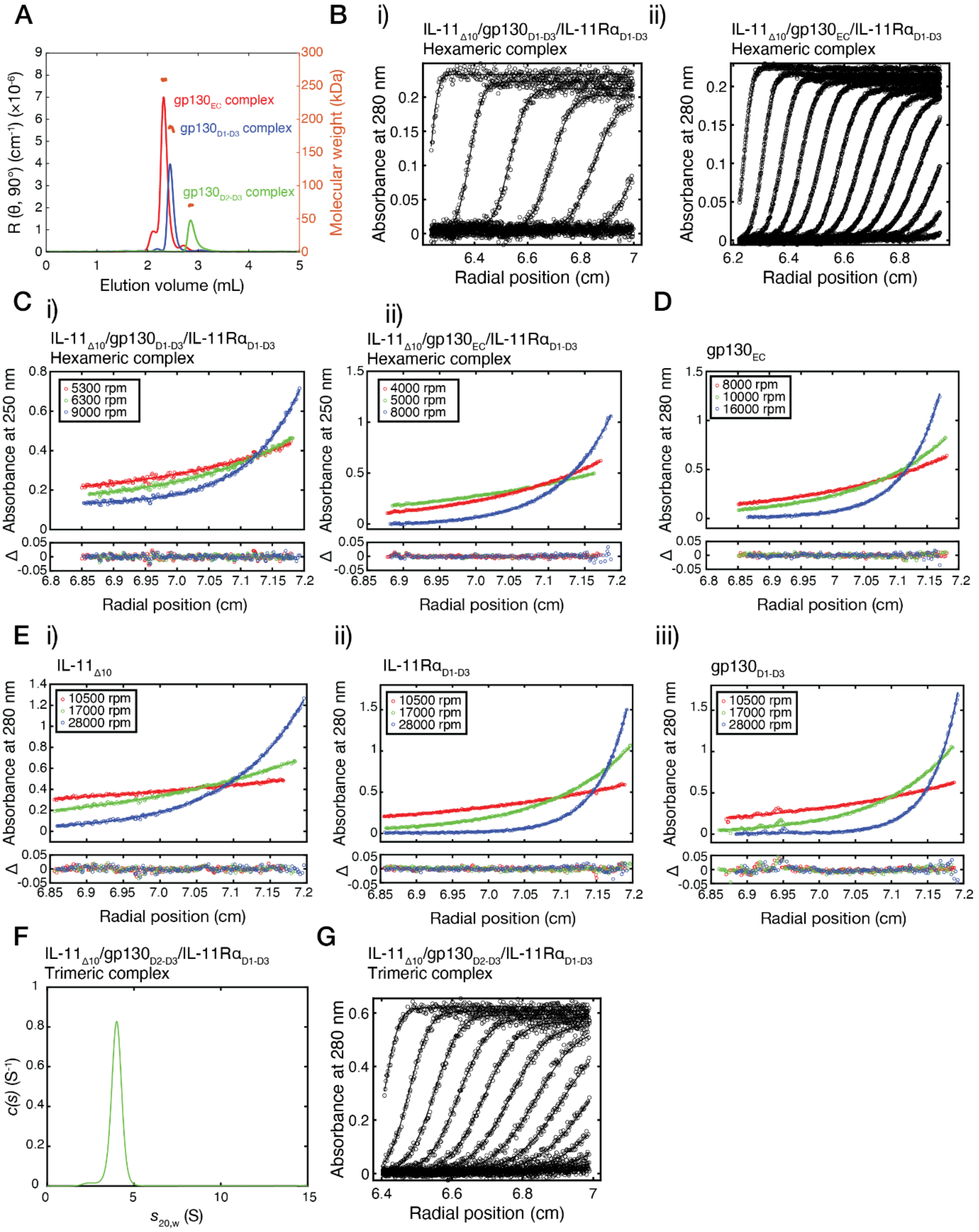

Supplementary Figure 5: MALS data, raw SV-AUC scans, and SE-AUC data related to Figure 1D, E. A) MALS data for the gp130$_{EC}$, gp130$_{D1\text{-}D3}$ and gp130$_{D2\text{-}D3}$ complexes. B) Raw SV-AUC scans for the data shown in Figure 1D for i) the gp130$_{D1\text{-}D3}$ complex, and ii) the gp130$_{EC}$ complex. C) SE-AUC data for i) the gp130$_{D1\text{-}D3}$ complex, and ii) the gp130$_{EC}$ complex. D) SE-AUC data for gp130$_{EC}$. E) SE-AUC data for individual components of the gp130$_{D1\text{-}D3}$ complex; i) IL-11$_{\Delta 10}$, ii) IL-11Rα$_{D1\text{-}D3}$, iii) gp130$_{D1\text{-}D3}$. F) Continuous sedimentation coefficient (c(s)) distribution for the complex formed between IL-11$_{\Delta 10}$, IL-11Rα$_{D1\text{-}D3}$, and gp130$_{D2\text{-}D3}$. G) Raw SV-AUC scans for the data shown in panel F.

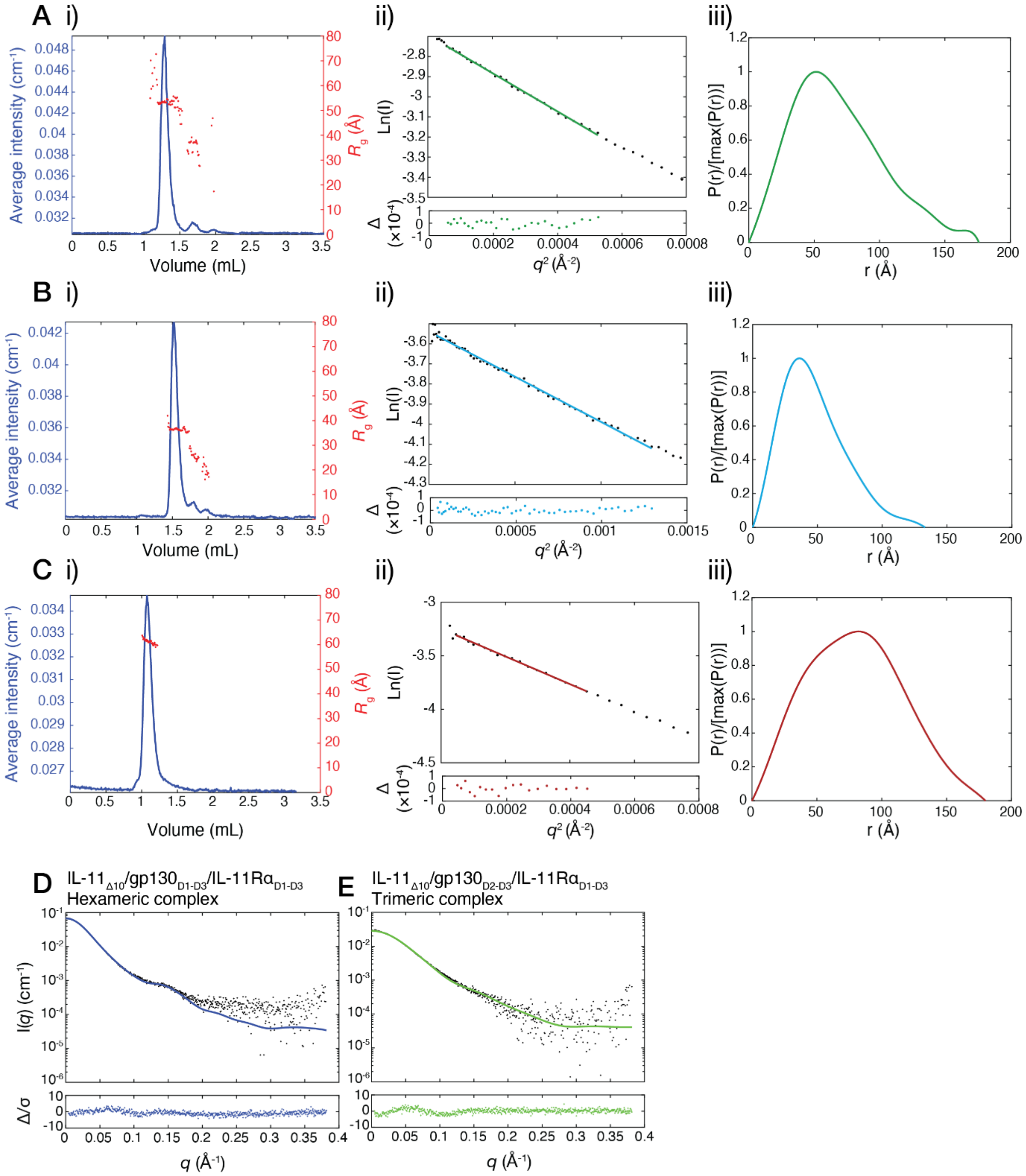

Supplementary Figure 6: Supplementary SAXS data, related to Figure 1E. A) Supplemental SAXS data for the gp130$_{D1\text{-}D3}$ complex; i) SEC-SAXS chromatogram, ii) Guinier plot, iii) pairwise distance distribution (P(r)) plot). B) Supplemental SAXS data for the gp130$_{D2\text{-}D3}$ complex; i) SEC-SAXS chromatogram, ii) Guinier plot, iii) pairwise distance distribution (P(r)) plot). C) Supplemental SAXS data for the gp130$_{EC}$ complex; i) SEC-SAXS chromatogram, ii) Guinier plot, iii) pairwise distance distribution (P(r)) plot). D) Fit of one hexamer from the IL-11 signalling complex crystal structure, to the scattering data from the gp130$_{D1\text{-}D3}$ complex (see Supplementary Table 3 for fitting statistics). E) Fit of the coordinates of the IL-11$_{\Delta10}$/IL-11Rα$_{D1\text{-}D3}$/gp130$_{D2\text{-}D3}$ complex (extracted from the atomic model of the IL-11$_{\Delta10}$/IL-11Rα$_{D1\text{-}D3}$/gp130$_{EC}$ complex) to the scattering data from the gp130$_{D2\text{-}D3}$ complex (see Supplementary Table 3).

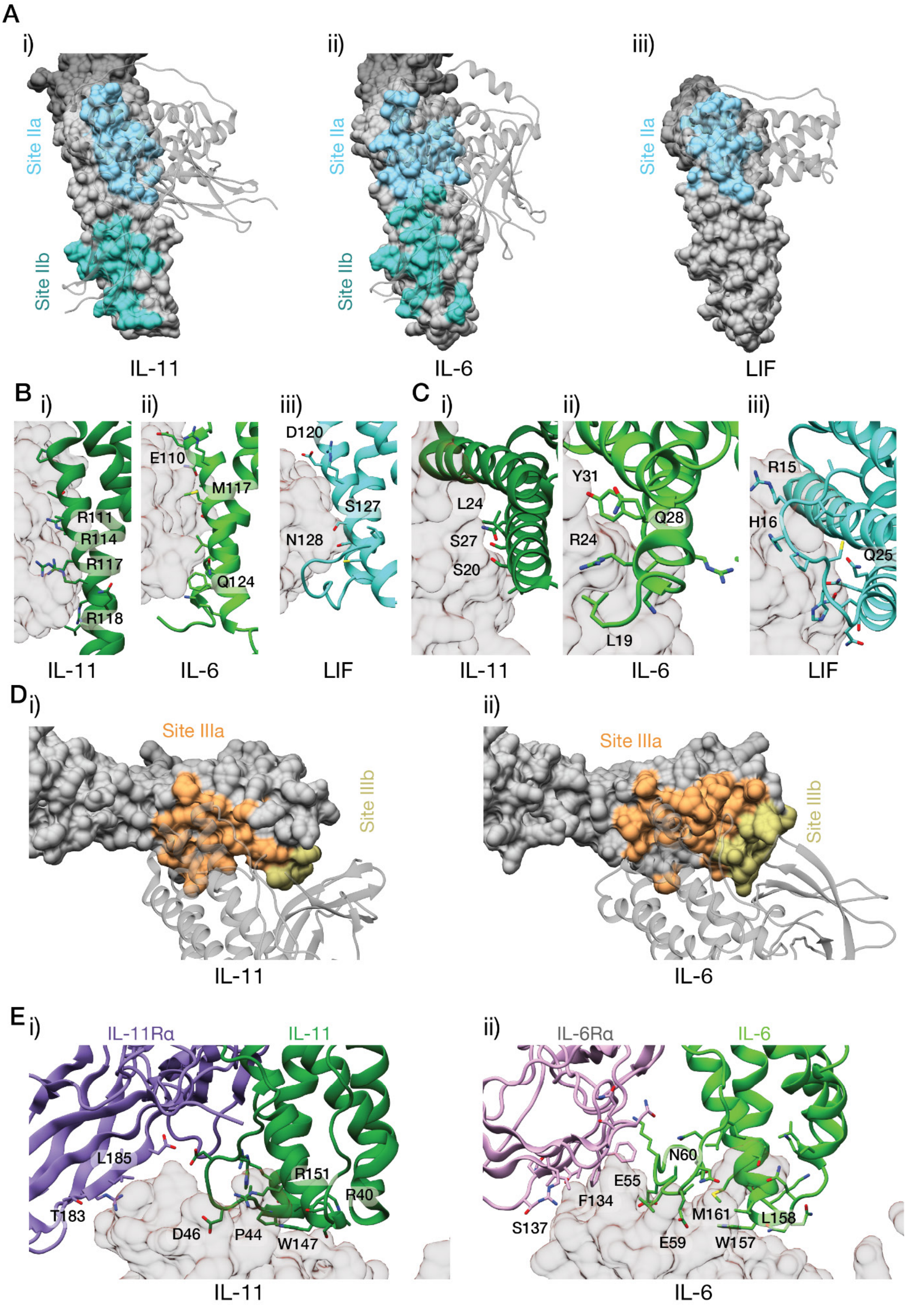

A
i)
ii)
iii)
Site IIa
Site IIb
IL-11
IL-6
LIF
B
C
D120
E110
M117
S127
N128
R111
R114
R117
R118
Q124
L24
S27
S20
Y31
Q28
R24
L19
R15
H16
Q25
D
Site IIIa
Site IIIb
E
IL-11Rα
IL-11
IL-6Rα
IL-6
L185
T183
D46
P44
W147
R151
R40
N60
E55
F134
S137
E59
M161
W157
L158

Supplementary Figure 7: Comparison of the IL-11 signalling complex with the IL-6[5] (PDB ID: 1P9M) and LIF[6] (PDB ID: 1PVH) signalling complexes. A) The site-II binding surface on gp130 for i) IL-11/IL-11Rα, ii) IL-6/IL-6Rα, iii) LIF. B) Interactions between gp130 and the C-helix of i) IL-11, ii) IL-6 and iii) LIF. C) Interactions between gp130 and the N-terminus of i) IL-11, ii) IL-6 and iii) LIF. D) Comparison of the site-III interfaces between i) IL-11/IL-11Rα and ii) IL-6/IL-6Rα. E) Molecular details of the site-III interface of i) IL-11/IL-11R and ii) IL-6/IL-6Rα.

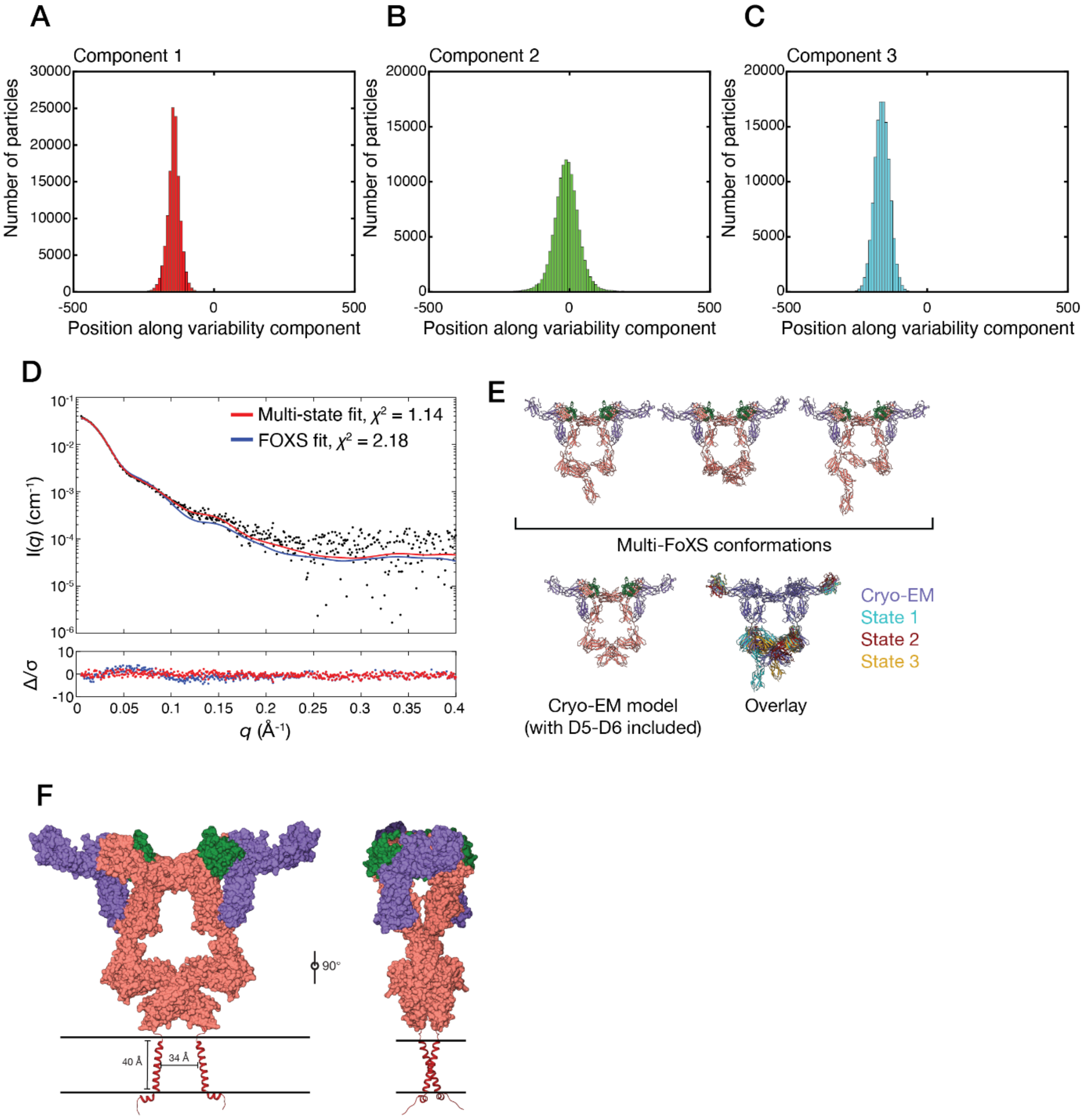

Supplementary Figure 8: Histograms indicating the number of particles along each variability component for, A) variability component 1, B) variability component 2, C) variability component 3. D) Fit of the gp130$_{EC}$ cryo-EM model with D5-D6 included, (blue line), and a three-state model with the IL-11Rα D1-D2, and the gp130 D3-D4, D4-D5, D5-D6 linkers flexible (red line; see Supplementary Table 3), to the gp130$_{EC}$ complex SAXS data. The fit of the cryo-EM model was generated using FoXS[7]. Multistate models were generated and fit using the Multi-FoXS server[8]. Note the same experimental SAXS data are presented in Figure 1E. E) Models fit to the SAXS data in panel D. F) Model of the membrane-bound IL-11 complex, generated using the Alphafold[9] prediction of full-length gp130 (AF-P40189-F1). The putative position of the cell membrane is indicated using two solid black lines.

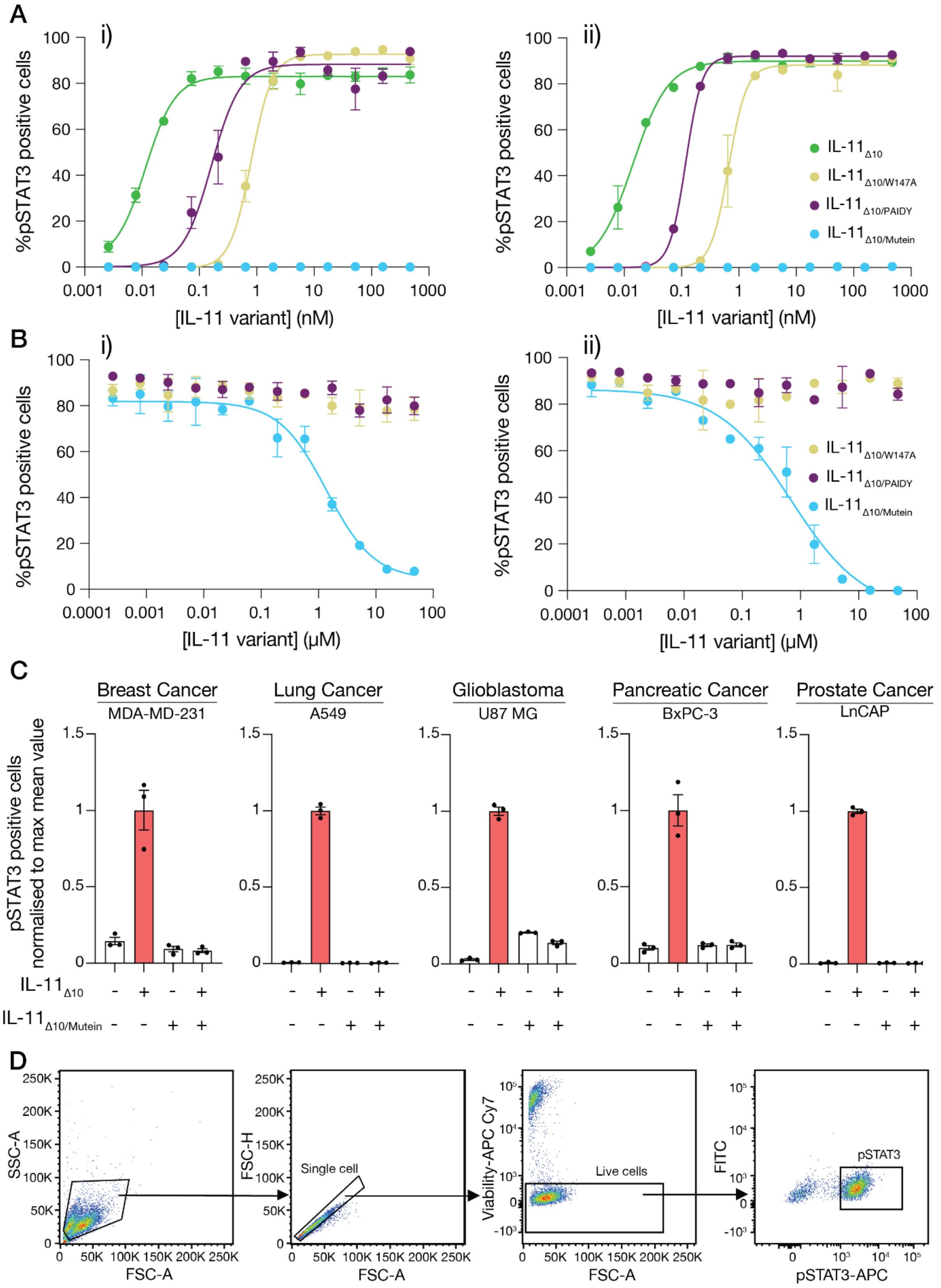

Supplementary Figure 9: Replicate experiments for biological assays and FACS gating strategy. A) Replicate dose-response curves for determination of the EC_{50} of IL-$11_{\Delta10}$ and other IL-11 variants. Data are presented as the mean ± SEM of three technical replicates. Independent experiments are shown in panels i and ii and Figure 4A. Total n = 3 independent

experiments. B) Replicate dose-response curves for determination of the IC_{50} of IL-$11_{\Delta10/Mutein}$. Data are presented as the mean ± SEM of three technical replicates. Independent experiments are shown in panels i and ii and Figure 4B. Total *n* = 3 independent experiments. Source data are provided as a Source Data file. C) Replicate experiment showing inhibition of IL-$11_{\Delta10}$ signalling by IL-$11_{\Delta10/Mutein}$ in the indicated human cancer cell lines. Data are presented as the mean ± SEM of three technical replicates. Independent experiment is shown in Figure 4C. Total of *n* = 2 independent experiments. D) Representative image of the flow cytometry gating strategy used to determine pSTAT3 positive cells data in Figure 4 and Supplementary Figure 9A-C. Obtained events were gated in FSC-A and SSC-A dot plot to eliminate debris. Single cells were gated on FSC-A and FSC-H to eliminate doublets. Viable cells were gated on an FSC-A vs Viability dye. pSTAT3 positive cells were gated on an pSTAT3 vs FITC (empty channel). FSC: Forward scatter, SSC: side scatter.

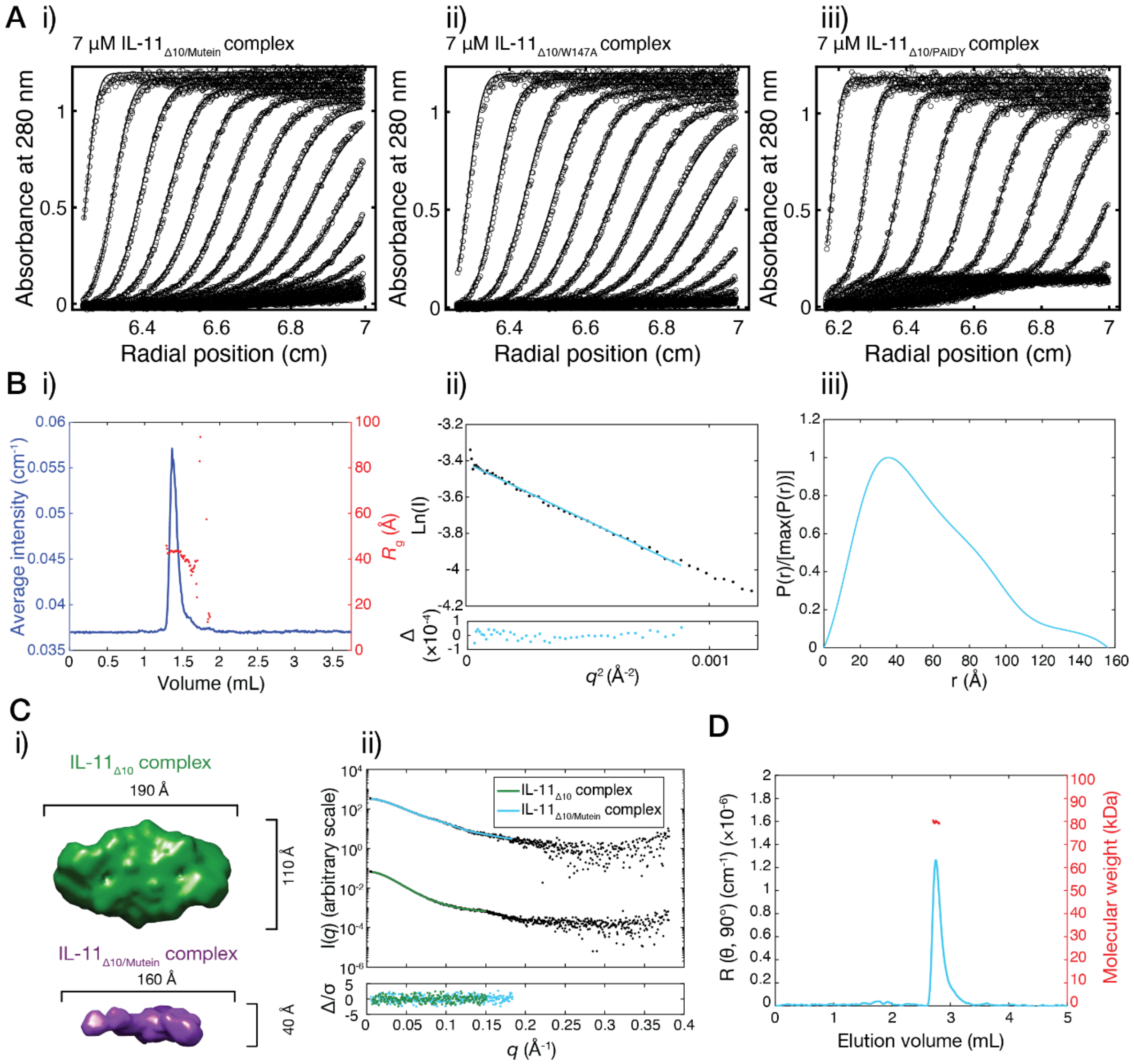

Supplementary Figure 10: Raw SV-AUC scans, supplementary SAXS and MALS data for Figure 5. A) Raw SV-AUC scans for the data shown in, i) Figure 5Ai, ii) Figure 5Aii, iii) Figure 5Aiii. B) Supplemental SAXS data for the IL-11$_{Δ10/Mutein}$ /IL-11Rα$_{D1-D3}$/gp130$_{D1-D3}$ complex; i) SEC-SAXS chromatogram, ii) Guinier plot, iii) pairwise distance distribution (P(r)) plot). C) *Ab inito* SAXS modelling of the complexes, calculated using *DAMMIN*[10], between IL-11$_{Δ10}$/IL-11$_{Δ10/Mutein}$, gp130$_{D1-D3}$ and IL-11Rα$_{D1-D3}$, i) shows the models, and ii) shows the fit to the raw scattering data. D) MALS data for the IL-11$_{Δ10/Mutein}$/IL-11Rα$_{D1-D3}$/gp130$_{D1-D3}$ complex.

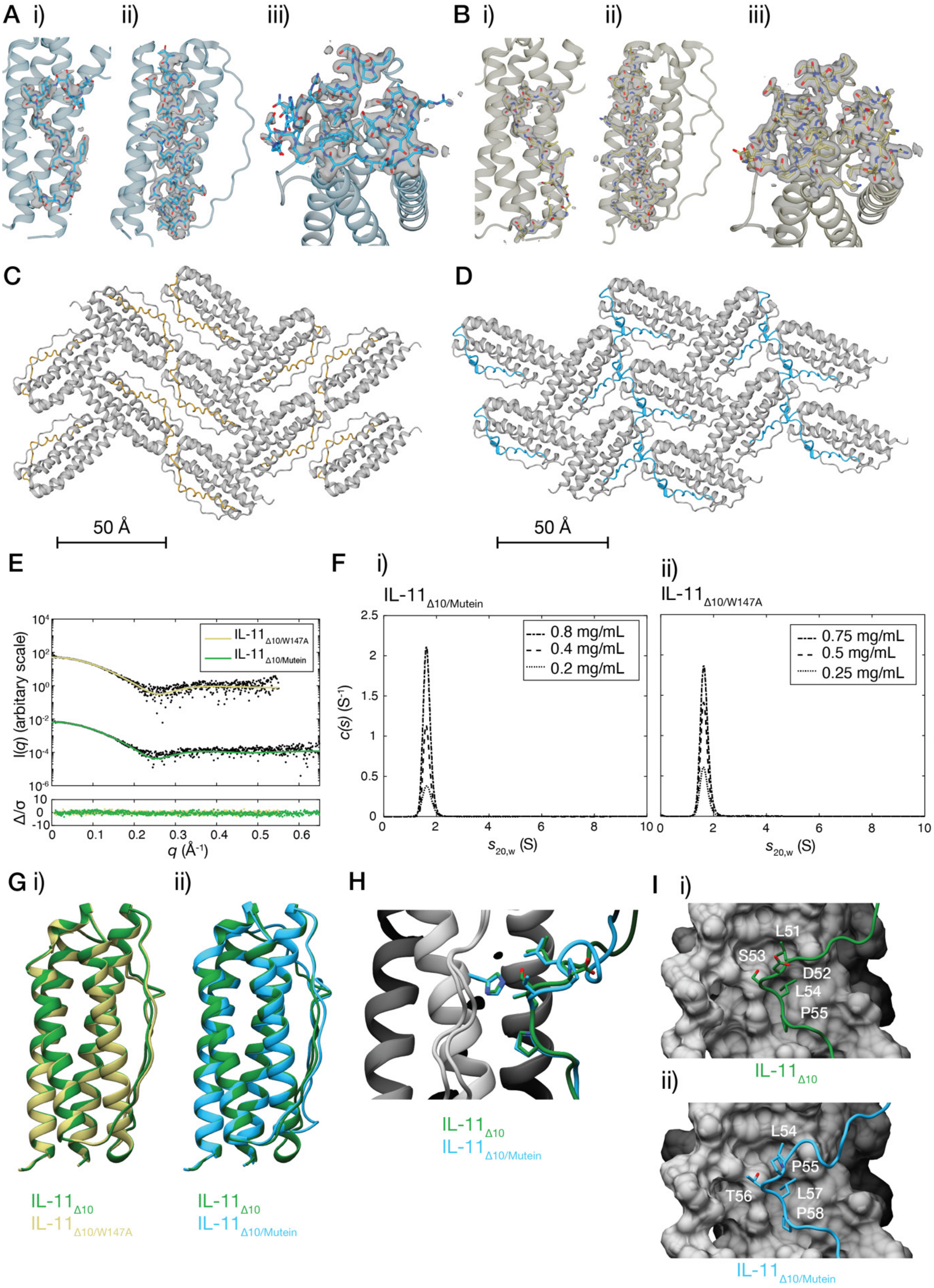

Supplementary Figure 11: Representative electron density, additional structural representations, and biophysical characterisation of IL-11$_{\Delta10/\text{Mutein}}$ and IL-11$_{\Delta10/\text{W147A}}$. A) Representative electron density for IL-11$_{\Delta10/\text{Mutein}}$; i) the AB loop, including the mutated PAIDY sequence, ii) helix C, iii) the site-III interface, including A147. B) Representative electron

density for IL-11$_{Δ10/W147A}$; i) the AB loop, including the AMSAG sequence, ii) helix C, iii) the site-III interface, including A147. C) Packing of IL-11$_{Δ10/W147A}$ in the crystal lattice, the AB loop is indicated in gold. D) Packing of IL-11$_{Δ10/Mutein}$ in the crystal lattice, the AB loop is indicated in blue. E) SAXS data collected on IL-11$_{Δ10/Mutein}$ and IL-11$_{Δ10/W147A}$, the fit shown is to the crystal structure coordinates. F) Continuous sedimentation coefficient (c(s)) distributions for i) IL-11$_{Δ10/Mutein}$ and ii) IL-11$_{Δ10/W147A}$, each measured at three concentrations. For raw scans, see Supplementary Figure 12. G) Overlay of the crystal structure of IL-11$_{Δ10}$ [11] (PDB ID: 6O4O) and the structure of i) IL-11$_{Δ10/W147A}$, ii) IL-11$_{Δ10/Mutein}$. H) Overlay of the Ser53/Thr56 region in IL-11$_{Δ10}$ and IL-11$_{Δ10/Mutein}$. I) Surface representation of the contacts between the AB loop and the α-helical core of i) IL-11$_{Δ10}$, ii) IL-11$_{Δ10/Mutein}$.

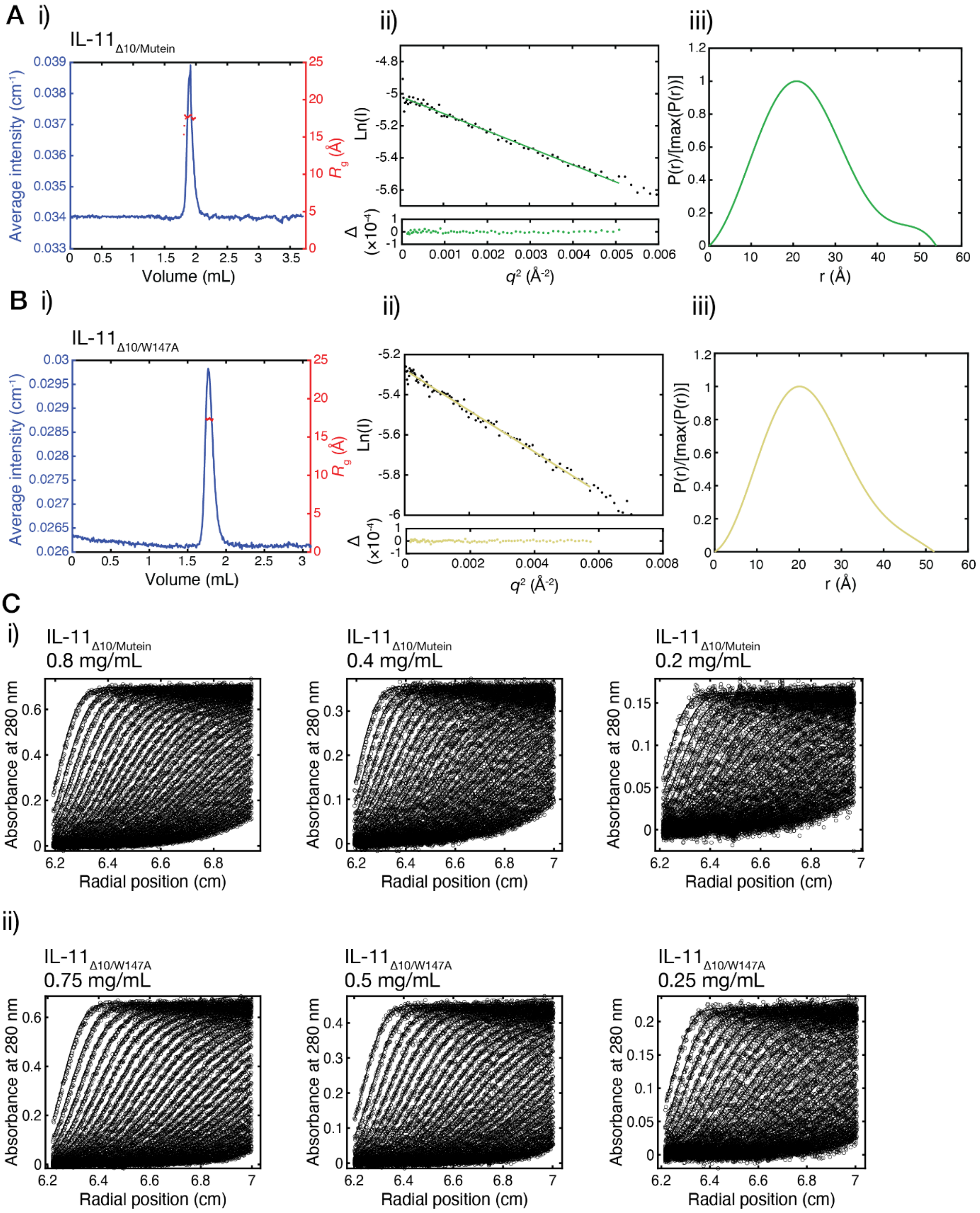

Supplementary Figure 12: Supplementary SAXS and AUC data, related to Supplementary Figure 11E, F. A) Supplemental SAXS data for IL-11$_{\Delta10/\text{Mutein}}$, i) SEC-SAXS chromatogram, ii) Guinier plot, iii) pairwise distance distribution (P(r)) plot). B) Supplemental SAXS data for IL-11$_{\Delta10/\text{W147A}}$, i) SEC-SAXS chromatogram, ii) Guinier plot, iii) pairwise distance distribution (P(r)) plot). C) Raw SV-AUC scans for the data shown in, i) Supplementary Figure 11Fi, ii) Supplementary Figure 11Fii.

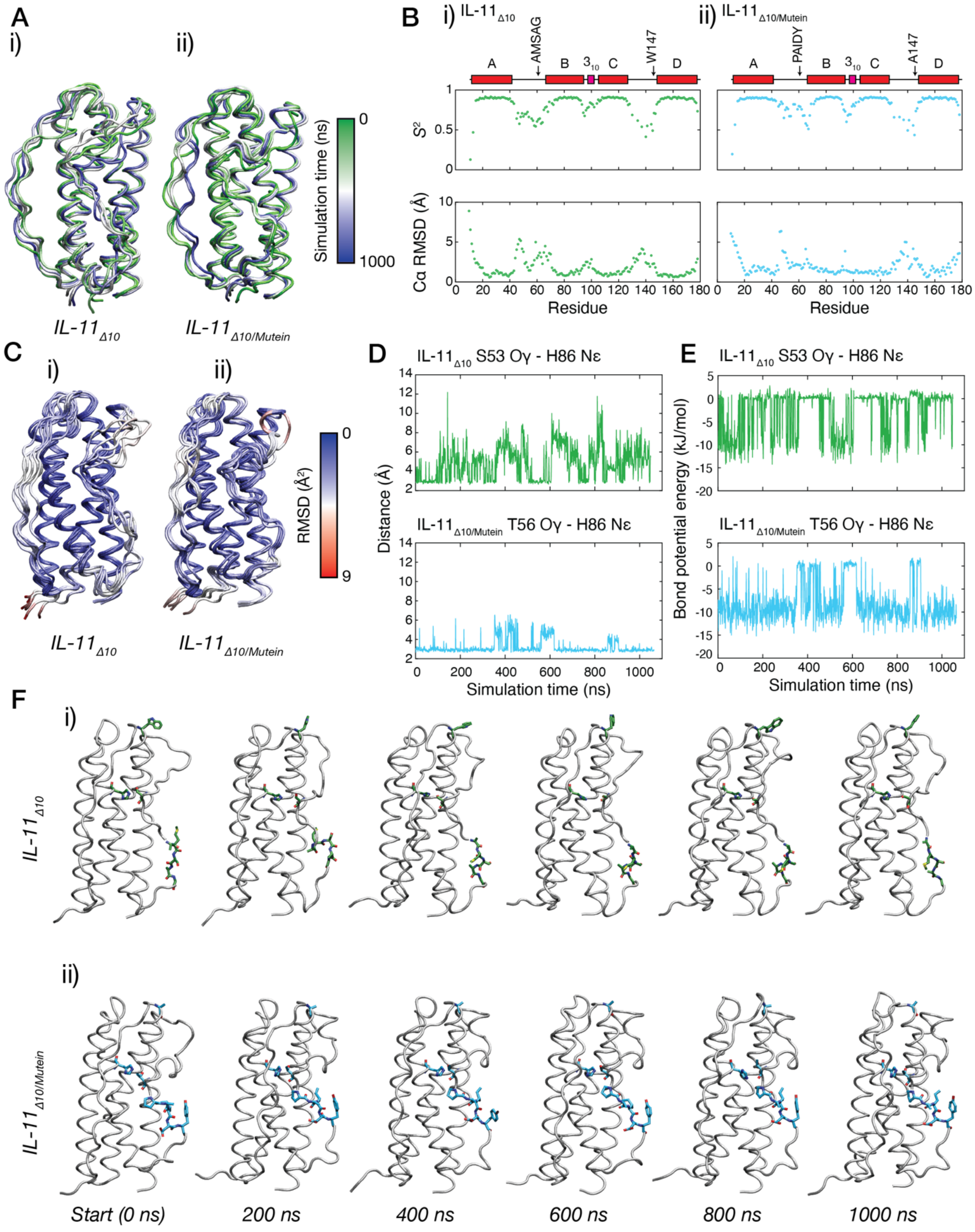

Supplementary Figure 13: Supplementary MD data, and DSF data. A) Overlay of frames from the 1 µs MD simulation of i) IL-11$_{\Delta10}$ and ii) IL-11$_{\Delta10/\text{Mutein}}$. Frames are shown at 200 ns intervals. B) Order parameter (S^2) and Cα RMSD values calculated from the 1 µs MD simulation of i) IL-11$_{\Delta10}$ and ii) IL-11$_{\Delta10/\text{Mutein}}$. A schematic representation of the secondary structure is shown above the plot, and the location of the mutations is indicated. C) Overlay of frames from the 1 µs MD simulation of i) IL-11$_{\Delta10}$ and ii) IL-11$_{\Delta10/\text{Mutein}}$, coloured by Cα RMSD. Frames are shown at 200 ns intervals. D) Distance between the γ oxygen of T56/S53 in IL-11$_{\Delta10}$ or IL-

$11_{\Delta10/Mutein}$ through a 1 µs MD simulation. E) Estimated hydrogen bond potential energy for the S/T Oγ and H Nε for IL-$11_{\Delta10}$ or IL-$11_{\Delta10/Mutein}$ through a 1 µs MD simulation. F) 200 ns snapshots of the MD simulations of i) IL-$11_{\Delta10}$ and ii) IL-$11_{\Delta10/Mutein}$. The AMSAG/PAIDY sequence, S53/T56, H86 and W147/A147 residues are displayed in the figure.

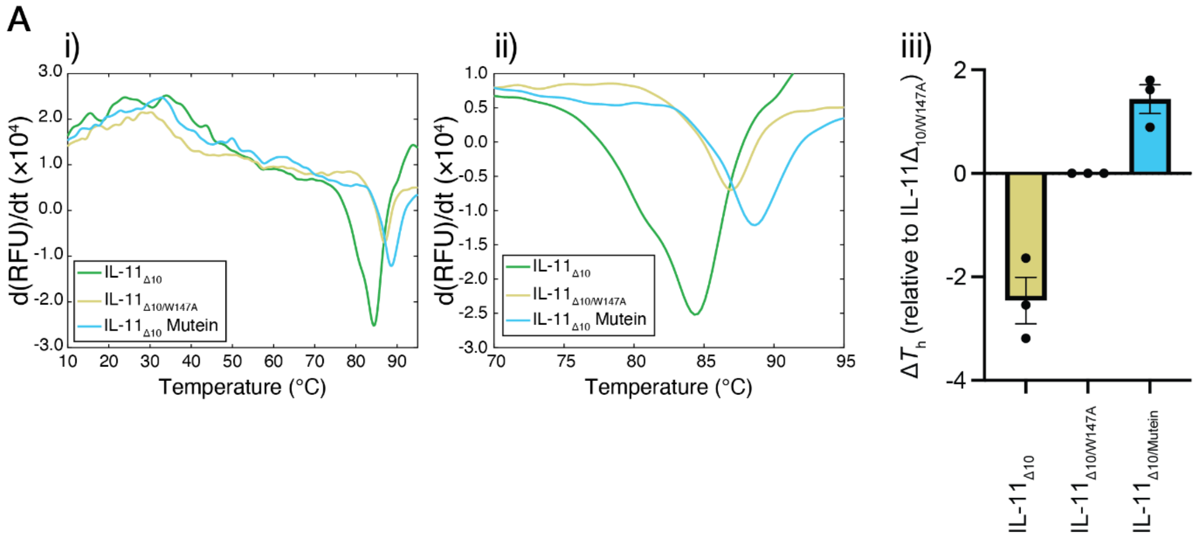

Supplementary Figure 14: Differential scanning fluorometry thermal melt data. A) Representative DSF first-derivative melt curve, i), with ii) highlighting the region of interest. iii) Bar graph, showing ΔT_h (relative to IL-11$_{\Delta 10/W147A}$). Data are presented as the mean ± SEM, n = 3 independent experiments. Source data are provided as a Source Data file.

Supplementary Tables:

Supplementary Table 1: Cryo-EM data collection, data processing and model building statistics.

	IL-11 signalling complex $gp130_{D1\text{-}D3}$/IL-$11_{\Delta 10}$/ IL-11Rα$_{D1\text{-}D3}$	*IL-11 signalling complex $gp130_{EC}$/IL-$11_{\Delta 10}$/ IL-11Rα$_{D1\text{-}D3}$*
**Data collection and image processing**		
Magnification	100,000	100,000
Electron energy (kV)	200	200
Electron exposure (e^-/Å^2)	52	52
Defocus range (µm)	0.8-2.0	0.8-2.0
Pixel size (Å)	1.31	1.31
Starting model	*De novo*	*De novo*
Symmetry imposed	C2	C2
Total number of micrographs	2010	6,861
Final particle images	204,455	125,373
Map resolution (Å)	3.5	3.76
FSC threshold	0.143	0.143
EMDB code	EMD-27641	EMD-27642
**Model building and refinement**		
Initial models used	PDB IDs 6O4O[11], 1I1R[12] (chain A), unpublished structure of IL-11Rα	PDB IDs 6O4O[11], 1I1R[12] (chain A), 3L5I[13], unpublished structure of IL-11Rα
Model resolution (Å)	3.9	4.1
FSC threshold	0.5	0.5
Sharpening *B* factor (Å^2)	151.24	130.98
Model composition		
Non-hydrogen atoms	10610	13550
Amino acid residues	1326	1714
Protein molecules	10	10
Real-space correlation		
CCvolume	0.72	0.72
CCmask	0.73	0.74
Mean *B* factor (Å^2)	54.86	72.88
RMS deviations		
Bond lengths (Å) (outliers > 4σ)	0.007 (0)	0.010 (0)
Bond angles (°) (outliers > 4σ)	1.386 (55)	1.383 (48)
Validation		
MolProbity score	2.16	2.33

Clashscore	18.79	23.97
Rotamer outliers (%)	0.52	0.00
CaBLAM outliers (%)	1.70	2.49
Cβ outliers	0.32	0.25
Ramachandran plot		
Favoured (%)	94.19	92.83
Allowed (%)	5.66	6.46
Outliers (%)	0.15	0.71
PDB code	8DPS	8DPT

Supplementary Table 2: X-ray crystallography data collection and refinement statistics.

	IL-11 signalling complex $gp130_{D1-D3}$/$IL\text{-}11_{FL}$/$IL\text{-}11R\alpha_{EC}$	*$IL11_{\Delta10/Mutein}$*	*$IL11_{\Delta10/W147A}$*
**Data collection**			
Space group	$P\ 3_1\ 1\ 2$	$P\ 2_1$	$P\ 2_1\ 2_1\ 2$
Wavelength (Å)	0.9537	0.9537	0.9537
Number of images	3600	3600	3600
Oscillation range per image (˚)	0.1	0.1	0.1
Detector	Eiger 16M	Eiger 16M	Eiger 16M
Cell dimensions			
a, *b*, *c* (Å)	163.41, 163.41, 506.62	27.23, 37.10, 68.53	38.51, 134.00, 27.09
α, β, γ (˚)	90, 90, 120	90, 101.31, 90	90, 90, 90
Resolution range for data processing (Å)	49.28-3.78 (4.20-3.78)	37.10-1.80 (1.84-1.80)	44.67-1.48 (1.51-1.48)
Resolution cut-offs from anisotropic analysis (Å)			
$0.894a^* - 0.447b^*$	4.93		
b^*	4.93		
c^*	3.58		
$R_{sym}^{\dagger}$	0.286 (2.522)	0.07 (1.167)	0.082 (1.859)
$R_{meas}^{\S}$	0.294 (2.584)	0.083 (1.374)	0.089 (2.003)
$R_{pim}^{\ddagger}$	0.064 (0.558)	0.043 (0.719)	0.034 (0.743)
$CC_{1/2}$	0.998 (0.548)	0.999 (0.603)	0.999 (0.590)
$I/\sigma(I)$	10.6 (1.5)	11.8 (1.4)	12.9 (1.3)
Total observations	947761 (48027)	85885 (5234)	313134 (16025)
Unique reflections	45270 (2262)	12637 (761)	24354 (1182)
Completeness (%)		100.0 (100.0)	100.0 (100.0)
Spherical (%)	58.1 (10.8)		

Ellipsoidal (%)	92.8 (72.0)		
Multiplicity	20.9 (21.2)	6.8 (6.9)	12.9 (13.6)
Wilson *B* factor (Å^2)	126.31	25.60	23.19
**Refinement**			
Resolution (Å)	40.86-3.78 (3.91-3.78)	33.61-1.80 (1.86-1.80)	37.02-1.48 (1.53-1.48)
Reflections used in refinement	43181 (296)	12627 (1241)	23081 (2232)
R_{free} reflections	2054 (21)	1173 (68)	1216 (129)
R_{work}	0.2805 (0.4323)	0.1978 (0.2899)	0.1889 (0.2732)
R_{free}	0.2966 (0.4378)	0.2306 (0.2773)	0.2185 (0.2696)
Protein molecules in asymmetric unit	18	1	1
Total nonhydrogen atoms	35652	1390	1429
Protein	35652	1304	1308
Ligand/ion	0	5	6
Solvent	0	71	114
Mean B *factor (Å^2)*	176.20	46.65	37.48
Protein	176.20	46.81	37.10
Ligand/ion	N/A	59.96	49.03
RMS deviations			
Bond lengths (Å) (outliers > 4σ)	0.005 (6)	0.003 (0)	0.005 (0)
Bond angles (°) (outliers > 4σ)	1.156 (86)	0.630 (0)	0.791 (0)
Rotamer outliers	0.16	0.73	0.00
Clashscore	14.16	4.14	4.10
Cβ outliers	0	0	0
Molprobity score	2.06	1.20	1.19
Ramachandran Plot			

Favoured (%)	94.10	98.20	98.80
Allowed (%)	5.22	1.80	1.20
Outliers (%)	0.68	0.0	0.0
PDB code	8DPU	8DPW	8DPV

† $R_{sym} = \sum_{hkl}\sum_{i} |I_i (hkl) \quad \langle I(hkl)\rangle \ | / \sum_{hkl}\sum_{i} I_i (hkl)$

§ $R_{meas} = \sum_{hkl} [N/(N \ \ 1)]^{½} \sum_{i} |I_i (hkl) \quad \langle I (hkl)\rangle \ | / \sum_{hkl} \sum_{i} I_i (hkl)$

‡ $R_{pim} = \sum_{hkl} [1/(N \ \ 1)]^{½} \sum_{i} |I_i (hkl) \quad \langle I (hkl)\rangle \ | / \sum_{hkl} \sum_{i} I_i (hkl)$

$CC_{1/2}$ = Pearson correlation coefficient between independently merged half datasets

Supplementary Table 3: SAXS data collection and refinement statistics.

	gp130$_{D1\text{-}D3}$ complex[a]	*gp130$_{EC}$ complex*[b]	*gp130$_{D2\text{-}D3}$ complex*[c]	*IL11$_{\Delta10/Mutein}$ complex*[d]	*IL11$_{\Delta10/Mutein}$*	*IL11$_{\Delta10/W147A}$*
**SAXS data collection**						
Instrument/source	Australian Synchrotron SAXS/WAXS beamline equipped with Pilatus 2M detector and sheathflow cell for SEC-SAXS[14, 15].					
Wavelength (Å)	1.078					
Beam energy (keV)	11.5					
Beam size (µm)	250 × 130					
Sample-to-detector distance (mm)	3538	2210	3538	3538	2038	2210
q measurement range (Å^{1}) [a]	0.004-0.38	0.005-0.55	0.004-0.38	0.004-0.38	0.007-0.664	0.005-0.55
Absolute scaling method	Comparison with scattering from 1 mm pure water					
Normalization	To transmitted intensity from beamstop counter					
Exposure time	1 s measurements from SEC-SAXS elution					
Sample temperature (K)	293					
**SEC-SAXS parameters**						
Column	Superdex 200 5/150 Increase					
Flow rate (mL/min)	0.45					0.4
Loading concentration (mg/mL)	2				5	
Injection volume (µL)	50					
Solvent	20 mM Tris-HCl pH 8.5, 150 mM NaCl, 0.2% sodium azide					
**Software employed**						
SAXS data reduction	*I*(*q*) vs *q* using Scatterbrain 2.8.2, SECSAXS solvent subtraction using *CHROMIXS* from *ATSAS* 2.8.3					

Basic analysis (Guinier, *P(r)*, molecular mass)	*PRIMUS* from *ATSAS* 2.8.3, *GNOM* from *ATSAS* 2.83					
Shape modelling	*DAMMIF* from *ATSAS* 2.8.3, *DAMAVER* from *ATSAS* 2.8.3, *DAMMIN* from *ATSAS* 2.8.3					
Calculation of theoretical intensities	*CRYSOL* from *ATSAS* 2.8.3, *FoXS and Multi-FoXS* web servers (accessed June 2023)					
Flexible fitting	*FoXS and Multi-FoXS* web servers (accessed June 2023)					
**Structural parameters**						
Mass from V_c (kDa) (expected mass, ratio to expected, in brackets) [b]	182.2 (169.8, 0.93)	290.0 (234.8, 0.81)	81.6 (73.6, 0.90)	87.9 (84.9, 0.96)	16.7 (18.2, 0.91)	16.7 (18.2, 0.91)
**Guinier analysis**						
R_g (Å)	53.10 ± 0.19	61.99 ± 0.46	36.49 ± 0.14	43.41 ± 0.18	17.82 ± 0.13	17.36 ± 0.12
I(0) (cm^{1})	$0.068 \pm 1.4\times10^{-4}$	$0.039 \pm 2.2\times10^{-4}$	$0.029 \pm 7\times10^{-5}$	$0.033 \pm 8.4\times10^{-5}$	$0.0066 \pm 2.6\times10^{-5}$	$0.0051 \pm 2.1\times10^{-5}$
qR_g min,max	0.41, 1.22	0.42, 1.32	0.24, 1.31	0.23, 1.29	0.20, 1.27	0.2, 1.32
P(r) analysis [c]						
R_g (Å)	54.08 ± 0.13	62.66 ± 0.37	37.52 ± 0.15	45.24 ± 0.19	17.80 ± 0.88	17.33 ± 0.69
I(0) (cm^{1})	$0.0677 \pm 1.2\times10^{-4}$	$0.03891 \pm 1.9\times10^{-4}$	$0.02908 \pm 6.8\times 10^{-5}$	$0.033 \pm 9.7\times10^{-5}$	$0.0066 \pm 2.2 \times 10^{-5}$	$0.0051 \pm 1.6 \times 10^{-5}$
D_{max} (Å)	176	210	133	156	54	52
Porod volume (Å^3)	404000	709000	127000	159000	20000	22200
**Shape modelling**						
DAMMIF (10 calculations, default parameters)						
q range for fitting (Å)	0.007-0.15			0.00054 – 0.18		

Symmetry, anisotropy assumptions	*P*2, none			*P*1, none		
Constant adjustment to intensities	5.11×10^{-4}			1.67×10^{-4}		
NSD (standard deviations)	1.281 (0.176)			1.028 (0.081)		
χ^2 range	1.114-1.129			0.9981.018		
Resolution (from *SASRES*[16]) (Å)	50 ± 4			47 ± 4		
DAMMIN (default parameters)						
q range for fitting (Å)	0.007-0.15			0.00054 – 0.18		
Symmetry, anisotropy assumptions	*P2*, none			*P*1, none		
χ^2	1.009			0.975		
Constant adjustment to intensities	5.03×10^{-4}			1.58×10^{-4}		
**Atomic modelling**						
CRYSOL (no constant subtraction)						
Structure	gp130$_{EC}$ complex (PDB ID: 8DPT) residues 2-300 chain A, D, chain B, E, chain C, F	gp130$_{EC}$ complex (PDB ID: 8DPT) with gp130 D5-D6 domains modelled	gp130$_{EC}$ complex (PDB ID: 8DPT) residues 100-300 chain A, chain B, chain C	gp130$_{EC}$ complex (PDB ID: 8DPT) residues 2-269 chain A, chain B, chain C	IL-11$_{\Delta 10/Mutein}$ crystal structure (PDB ID: 8DPW)	IL-11$_{\Delta 10/W147A}$ crystal structure (PDB ID: 8DPV)
χ^2	3.06	2.66	1.76	1.72	1.37	0.91
Calculated R_g (Å)	53.38	61.04	36.12	43.30	17.61	17.39
Structure	IL-11$_{\Delta 10}$ complex crystal structure					

	(PDB ID: 8DPU) chains A-F					
χ^2	2.32					
Calculated R_g (Å)	54.00					
FoXS						
Structure		$gp130_{EC}$ complex (PDB ID: 8DPT) with gp130 D5-D6 domains modelled				
χ^2		2.18				
Calculated R_g (Å)		59.32				
Multi-FoXS						
Structure		$gp130_{EC}$ complex (PDB ID: 8DPT) with gp130 D5-D6 domains modelled				
Flexible residues		$gp130_{EC}$: 304-305; 395-396; 496-497 IL-11Rα: 90-91				
χ^2		1.14				
Calculated R_g range (Å)		60.3-61.8				
SASBDB code	SASDLM3	SASDLN3	SASDLP3	SASDLS3	SASDLR3	SASDLQ3

[a] Hexameric complex between $gp130_{D1\text{-}D3}$/$IL\text{-}11_{\Delta10}$/$IL\text{-}11R\alpha_{D1\text{-}D3}$

[b] Hexameric complex between $gp130_{EC}$/$IL\text{-}11_{\Delta10}$/$IL\text{-}11R\alpha_{D1\text{-}D3}$

[c] Trimeric complex between $gp130_{D2\text{-}D3}$/$IL\text{-}11_{\Delta10}$/$IL\text{-}11R\alpha_{D1\text{-}D3}$

[d] Trimeric complex between $gp130_{D1\text{-}D3}$/$IL\text{-}11_{\Delta10/Mutein}$/$IL\text{-}11R\alpha_{D1\text{-}D3}$

Supplementary Table 4: Complete ITC thermodynamic parameters. Values without brackets are provided in units of joules, values in square brackets are provided in units of calories. Errors are ± standard error, n = 3 independent titrations for all experiments. Summary statistics are shown. Source data are provided in the Source Data file.

Binding partners		K_D, nM	ΔH, kJ/mol [kcal/mol]	ΔS, J/mol K [cal/mol K]	ΔG, kJ/mol [kcal/mol]	Incompetent fraction [a]	T (K)	-TΔS, kJ/mol [kcal/mol]
IL-$11_{Δ10}$/IL-$11Rα_{D1-D3}$	$gp130_{D1-D3}$	3 ± 2	-34 ± 0.4 [-8.2 ± 0.1]	51 ± 9.4 [12 ± 2.3]	-49 ± 3.0 [-12 ± 0.7]	0.32 ± 0.008	288	-14 [-3.5]
IL-$11_{Δ10}$/IL-$11Rα_{D1-D3}$	$gp130_{D2-D3}$	380 ± 190	27 ± 2.0 [6.5 ± 0.5]	220 ± 4.2 [53 ± 1.0]	-36 ± 1.7 [-8.7 ± 0.4]	0.25 ± 0.03	288	-63 [-15]
IL-$11_{Δ10}$/IL-$11Rα_{D1-D3}$	$gp130_{EC}$	4 ± 2	-21 ± 0.7 [-5.0 ± 0.2]	95 ± 5.5 [22 ± 1.3]	-49 ± 1.2 [-11 ± 0.3]	0.36 ±0.03	288	-28 [-6.8]
IL-$11Rα_{D1-D3}$	IL-$11_{Δ10/W147A}$	10 ± 8	-22 ± 0.6 [-5.3 ± 0.1]	87 ± 11 [20 ± 2.6]	-48 ± 2.7 [-11 ± 0.7]	0.08 ± 0.04	303	-26 [-6.3]
IL-$11Rα_{D1-D3}$	IL-$11_{Δ10/Mutein}$	38 ± 9.4	-25 ± 1.1 [-5.9 ± 0.3]	64 ± 4.1 [15 ± 1.0]	-43 ± 0.6 [-10.3 ± 0.1]	0.04 ± 0.02	303	-18 [-4.3]
IL-$11Rα_{D1-D3}$	IL-$11_{Δ10/PAIDY}$	81 ± 44	-30 ± 1.3 [-7.2 ± 0.3]	38 ± 11.2 [9.2 ± 2.7]	-42 ± 1.4 [-10.0 ± 0.3]	0.15 ± 0.05	303	-11 [-2.8]
IL-$11_{Δ10/Mutein}$/ IL-$11Rα_{D1-D3}$	$gp130_{D1-D3}$	55 ± 4.1	22 ± 0.7 [5.4 ± 0.2]	220 ± 1.9 [51 ± 0.5]	-40 ± 0.2 [-9.6 ± 0.04]	0.43 ± 0.03	288	-62 [-15]
IL-$11_{Δ10/W147A}$/ IL-$11Rα_{D1-D3}$	$gp130_{D2-D3}$	130 ± 14	24 ± 0.7 [5.7 ± 0.2]	210 ± 2.6 [51 ± 0.6]	-38 ± 0.2 [-9.1 ± 0.05]	0.33 ± 0.03	288	-62 [-15]
IL-$11_{Δ10/PAIDY}$/ IL-$11Rα_{D1-D3}$	$gp130_{D2-D3}$	60 ± 16	23 ± 0.8 [5.5 ± 0.2]	220 ± 2.5 [52.2 ± 0.6]	-40 ± 0.7 [-9.6 ± 0.2]	0.35 ± 0.02	288	-63 [-15]
IL-$11_{Δ10/PAIDY}$/ IL-$11Rα_{D1-D3}$	$gp130_{D1-D3}$	20 ± 17	-31 ± 3.0 [-7.4 ± 0.7]	51 ± 6.4 [12 ± 1.5]	-47 ± 2.3 [-11 ± 0.5]	0.34 ± 0.07	288	-16 [-3.7]

[a] Similar to (1-N), see refs [17, 18].

Supplementary Table 5: Complete SPR kinetic parameters, n = 2 experiments, errors shown are ± standard error. Summary statistics are shown. Source data are provided in the Source Data file.

		K_D (nM)	k_d (× 10^{-1} s^{-1})	k_a (× 10^6 M^{-1} s^{-1})	T (K)
$IL11_{Δ10}$ - Avitag	IL-11R$α_{D1-D3}$	78 ± 1	1.1 ± 0.2	1.4 ± 0.2	298
$IL11_{Δ10/Mutein}$ - Avitag	IL-11R$α_{D1-D3}$	33 ± 3	0.34 ± 0.01	1.0 ± 0.1	298

Supplementary Discussion

Gp130 is a receptor shared by most other members of the IL-6 family of cytokines. Structures have been solved of the hexameric IL-6 signalling complex[5], which forms analogous site-II and site-III interactions with gp130, and the LIF/gp130 complex, which forms an analogous site-II interaction with gp130[6] (Supplementary Figure 7). The LIF and IL-6 gp130 site-II interactions have been previously compared[6]. IL-11, IL-6 and LIF interact with a similar surface on gp130 (Supplementary Figure 7A), with major common contacts are formed by residues 142-147 and 165-171 of gp130, the N-terminal region of the cytokine and B helix of the cytokine. F163 of gp130 forms the major contact to the N-terminal end of the helix of the cytokine. The cytokine B-helix contacts a similar region of gp130 in the three structures, however in IL-11 the contacts are more extensive, a consequence of the additional surface area buried by arginine residues 111, 114, 117 and 118 in IL-11 (Supplementary Figure 7Bi). The contacts with helix B of IL-6 and LIF are dominated by a number of small hydrophobic residues (Supplementary Figure 7Bii-iii) and are overall less extensive compared to IL-11. The N-terminal region of IL-11, IL-6 and LIF also interact with gp130 (Supplementary Figure 7C). The N-terminus of LIF forms more extensive contacts with gp130 compared to IL-6 and IL-11, which may underpin the ability of LIF to interact with gp130 without first interacting with an α-receptor[6] (Supplementary Figure 7Cii). The interaction between IL-6 and IL-11 with gp130 is complimented by an additional site-IIB interface between the α-receptor and gp130. In both complexes, the surface bound on gp130 is very similar (Supplementary Figure 7A), with the binding surface predominantly formed by residues 250-265 of gp130. Notably, the interaction between IL-11Rα and gp130 is more electrostatic in character and results in formation of ten hydrogen bonds, compared to five for the IL-6Rα/gp130 complex.

IL-6 and IL-11 both form an additional interaction with D1 of gp130 at site-III to form the hexameric signalling complex. LIF forms an analogous interaction with D4 of LIFR[19]. The IL-6/gp130 site-III interaction is more extensive compared to the IL-11/gp130 site-III interaction (Supplementary Figure 7D-E). The surface bound on D1 of gp130 is similar between the two cytokines (Supplementary Figure 7D). In both interactions, the predominant contacts are made by the N-terminal end of the AB loop and helix D of the cytokine, the N-terminus of gp130, and residues 90-98 of gp130. IL-6 makes more extensive contacts between the N-terminal end of the AB loop and the N-terminus of gp130 (Supplementary Figure 7Eii). Analogous interactions are not present in the IL-11 complex; indeed, the N-terminal loop of gp130 is poorly resolved in all of our density maps. An analogous tryptophan (W147 in IL-11, W157 in IL-6), at the N-terminal end of helix D forms a key site-III contact in both complexes (Supplementary Figure 7E). Additional hydrophobic residues present in the IL-6 complex at the end of helix D (e.g. L158) that are not present in the IL-11 complex. Similarly, the site-IIIb interface is significantly less extensive in the IL-11 complex, compared to the IL-6 complex (Supplementary Figure 7E). In both complexes the major contact between gp130 and the α-receptor is formed by a short loop in gp130 (residues 86-89), however in the IL-6 complex these contacts are more extensive, and additional contacts are contributed by an additional short loop of gp130 (residues 35-37). The altered pose of IL-11Rα compared to IL-6Rα in the complex appears to have reduced the contacts formed between IL-11Rα and gp130 at site-IIIb (Supplementary Figure 7E). Overall, the IL-11 complex structure reinforces a previous suggestion[6] that gp130 has evolved to interact with unique and structurally diverse cytokines, in both the CHR and D1 of gp130.

Supplementary References

1. Punjani A, Rubinstein JL, Fleet DJ, Brubaker MA. CryoSPARC: Algorithms for rapid unsupervised cryo-EM structure determination. *Nature Methods* **14**, 290-296 (2017).

2. Adams PD*, et al.* PHENIX: A comprehensive Python-based system for macromolecular structure solution. *Acta Crystallographica Section D: Biological Crystallography* **66**, 213-221 (2010).

3. Afonine PV*, et al.* New tools for the analysis and validation of Cryo-EM maps and atomic models. *Acta Crystallographica Section D Structural Biology* **74**, 814-840 (2018).

4. Kucukelbir A, Sigworth FJ, Tagare HD. Quantifying the local resolution of cryo-EM density maps. *Nature Methods* **11**, 63-65 (2014).

5. Boulanger MJ, Chow D-c, Brevnova EE, Garcia KC. Hexameric structure and assembly of the interleukin-6/IL-6 alpha-receptor/gp130 complex. *Science* **300**, 2101-2104 (2003).

6. Boulanger MJ, Bankovich AJ, Kortemme T, Baker D, Garcia KC. Convergent mechanisms for recognition of divergent cytokines by the shared signaling receptor gp130. *Molecular Cell* **12**, 577-589 (2003).

7. Schneidman-Duhovny D, Hammel M, Tainer JA, Sali A. Accurate SAXS profile computation and its assessment by contrast variation experiments. *Biophys J* **105**, 962-974 (2013).

8. Schneidman-Duhovny D, Hammel M, Tainer JA, Sali A. FoXS, FoXSDock and MultiFoXS: Single-state and multi-state structural modeling of proteins and their complexes based on SAXS profiles. *Nucleic Acids Res* **44**, W424-429 (2016).

9. Jumper J*, et al.* Highly accurate protein structure prediction with AlphaFold. *Nature* **596**, 583-589 (2021).

10. Svergun D. Restoring low resolution structure of biological macromolecules from solution scattering using simulated annealing. *Biophysical Journal* **76**, 2879-2886 (1999).

11. Metcalfe RD*, et al.* The structure of the extracellular domains of human interleukin 11alpha receptor reveals mechanisms of cytokine engagement. *J Biol Chem* **295**, 8285-8301 (2020).

12. Chow D-C, He X, Snow aL, Rose-John S, Garcia K. Structure of an extracellular gp130 cytokine receptor signaling complex. *Science* **291**, 2150-2155 (2001).

13. Xu Y*, et al.* Crystal structure of the entire ectodomain of gp130: Insights into the molecular assembly of the tall cytokine receptor complexes. *Journal of Biological Chemistry* **285**, 21214-21218 (2010).

14. Ryan TM*, et al.* An optimized SEC-SAXS system enabling high X-ray dose for rapid SAXS assessment with correlated UV measurements for biomolecular structure analysis:. *Journal of Applied Crystallography* **51**, 97-111 (2018).

15. Kirby N*, et al.* Improved radiation dose efficiency in solution SAXS using a sheath flow sample environment. *Acta Crystallographica Section D Structural Biology* **72**, 1254-1266 (2016).

16. Tuukkanen AT, Kleywegt GJ, Svergun DI. Resolution of ab initio shapes determined from small-angle scattering *IUCrJ* **3**, 440-447 (2016).

17. Zhao H, Schuck P. Combining biophysical methods for the analysis of protein complex stoichiometry and affinity in SEDPHAT. *Acta Crystallographica Section D: Biological Crystallography* **71**, 3-14 (2015).

18. Scheuermann TH, Brautigam CA. High-precision, automated integration of multiple isothermal titration calorimetric thermograms: new features of NITPIC. *Methods* **76**, 87-98 (2015).

19. Huyton T*, et al.* An unusual cytokine:Ig-domain interaction revealed in the crystal structure of leukemia inhibitory factor (LIF) in complex with the LIF receptor. *Proceedings of the National Academy of Sciences* **104**, 12737-12742 (2007).
