# Supplementary material for: Structures of the interleukin 11 signalling complex reveal gp130 dynamics and the inhibitory mechanism of a cytokine variant
Source: Nat Commun. 2023 Nov 20;14:7543. doi: 10.1038/s41467-023-42754-w (PMC10662374; doi:10.1038/s41467-023-42754-w)
Supplement: Supplementary file 2 — Description of Additional Supplementary Files [file 41467_2023_42754_MOESM2_ESM.pdf]

Description of Additional Supplementary Files

File Name: Supplementary Movie 1
Description: Movie showing variability component 1 for the $gp130_{EC}$ complex.

File Name: Supplementary Movie 2
Description: Movie showing variability component 2 for the $gp130_{EC}$ complex.

File Name: Supplementary Movie 3
Description: Movie showing variability component 3 for the $gp130_{EC}$ complex.

File Name: Supplementary Movie 4
Description: Animation of 1 µs MD simulation of IL-$11_{\Delta10}$. The first half of the movie shows an overview of the protein through the simulation, the second half focuses on the S53 Oγ/H86 Nε hydrogen bond.

File Name: Supplementary Movie 5
Description: Animation of 1 µs MD simulation of IL-$11_{\Delta10/Mutein}$. The first half of the movie shows an overview of the protein through the simulation, the second half focuses on the T56 Oγ/H86 Nε hydrogen bond.
