# Supplementary material for: Structures of the interleukin 11 signalling complex reveal gp130 dynamics and the inhibitory mechanism of a cytokine variant
Source: Nat Commun. 2023 Nov 20;14:7543. doi: 10.1038/s41467-023-42754-w (PMC10662374; doi:10.1038/s41467-023-42754-w)
Supplement: Supplementary file 8 — Reporting Summary [file 41467_2023_42754_MOESM8_ESM.pdf]

nature portfolio

Corresponding author(s): Michael Griffin

Last updated by author(s): Oct 18, 2023

Reporting Summary

Nature Portfolio wishes to improve the reproducibility of the work that we publish. This form provides structure for consistency and transparency in reporting. For further information on Nature Portfolio policies, see our Editorial Policies and the Editorial Policy Checklist.

Statistics

For all statistical analyses, confirm that the following items are present in the figure legend, table legend, main text, or Methods section.

n/a	Confirmed	
☐	☒	The exact sample size (*n*) for each experimental group/condition, given as a discrete number and unit of measurement
☐	☒	A statement on whether measurements were taken from distinct samples or whether the same sample was measured repeatedly
☐	☒	The statistical test(s) used AND whether they are one- or two-sided<br>*Only common tests should be described solely by name; describe more complex techniques in the Methods section.*
☒	☐	A description of all covariates tested
☒	☐	A description of any assumptions or corrections, such as tests of normality and adjustment for multiple comparisons
☐	☒	A full description of the statistical parameters including central tendency (e.g. means) or other basic estimates (e.g. regression coefficient) AND variation (e.g. standard deviation) or associated estimates of uncertainty (e.g. confidence intervals)
☒	☐	For null hypothesis testing, the test statistic (e.g. *F*, *t*, *r*) with confidence intervals, effect sizes, degrees of freedom and *P* value noted<br>*Give P values as exact values whenever suitable.*
☒	☐	For Bayesian analysis, information on the choice of priors and Markov chain Monte Carlo settings
☒	☐	For hierarchical and complex designs, identification of the appropriate level for tests and full reporting of outcomes
☐	☒	Estimates of effect sizes (e.g. Cohen's *d*, Pearson's *r*), indicating how they were calculated

Our web collection on statistics for biologists contains articles on many of the points above.

Software and code

Policy information about availability of computer code

Data collection	EPU 1.9 software on FEI Talos Arctica, Scatterbrain v1.0
Data analysis	Relion 3.0, Cryosparc 2.1, MotionCor 2.1, Phenix v.1.19.2 (including phenix.real_space_refine, phenix_refine, phenix.validation_cryoEM, phenix_autobuild, EMRinger, MOLProbity), XDS build 20170923, CCP4 v. 7.0.045 (incorporating (POINTLESS v. 1.11.4, AIMLESS v.0.5.32), PHASER v.2.7.16, COOT v0.9.6, SEDFIT v16.1c, SEDNTERP v20130519, SEDPHAT v15.2b, Scatterbrain v1.0, ATSAS v. 3.0.2 (incorporating CHROMIXS, CRYSOL v.0.28, DAMMIF v. 1.1.2 DAMMIN v. 5.3, DAMAVER v.5.0), NAMD 2.1.3b1, ASTRA v.7.3.2.19, CHARMM22, UCSF Chimera v1.16, USCF ChimeraX v.1.5, VMD 1.9.3, FloJo 10.8.2, Pymol 2.2, GraphPad Prism 9.4.1, STARANISO v. 1.0.4, Multi-FoXS v. main.d5bb161

For manuscripts utilizing custom algorithms or software that are central to the research but not yet described in published literature, software must be made available to editors and reviewers. We strongly encourage code deposition in a community repository (e.g. GitHub). See the Nature Portfolio guidelines for submitting code & software for further information.

Data

Policy information about availability of data

All manuscripts must include a data availability statement. This statement should provide the following information, where applicable:

- Accession codes, unique identifiers, or web links for publicly available datasets
- A description of any restrictions on data availability
- For clinical datasets or third party data, please ensure that the statement adheres to our policy

Cryo-EM maps generated in this study have been deposited in the Electron Microscopy Data Bank (EMDB) under accession codes EMD-27641 [https://www.ebi.ac.uk/pdbe/entry/emdb/EMD-27641] (IL-11Δ10/IL-11RαD1-D3/gp130D1-D3 complex), EMD-27632 [https://www.ebi.ac.uk/pdbe/entry/emdb/EMD-27642] (IL-11Δ10/IL-11RαD1-D3/gp130EC complex). Atomic model coordinates generated in this study have been deposited in the Protein Data Bank (PDB) under accession codes 8DPS [http://doi.org/10.2210/pdb8DPS/pdb] (IL-11Δ10/IL-11RαD1-D3/gp130D1-D3 complex), 8DPT [http://doi.org/10.2210/pdb8DPT/pdb] (IL-11Δ10/IL-11RαD1-D3/gp130EC complex). Structure factors and atomic model coordinates generated in this study have been deposited in the PDB under accession codes 8DPU [http://doi.org/10.2210/pdb8DPU/pdb] (IL-11Δ10/IL-11RαD1-D3/gp130D1-D3 complex), 8DPV [http://doi.org/10.2210/pdb8DPV/pdb] (IL-11Δ10/W147A), 8DPW [http://doi.org/10.2210/pdb8DPW/pdb] (IL-11Δ10/Mutein).

Previously published coordinates used in this study are available in the PDB under accession codes 6O4O [http://doi.org/10.2210/pdb6O4O/pdb] (IL-11Δ10), 1I1R [http://doi.org/10.2210/pdb1I1R/pdb] (vIL-6 signaling complex), 3L5I [http://doi.org/10.2210/pdb1I1R/pdb] (gp130D4-D6), 4MHL [http://doi.org/10.2210/pdb1I1R/pdb] (IL-11), 1P9M [http://doi.org/10.2210/pdb1I1R/pdb] (IL-6 signaling complex) and 1PVH [http://doi.org/10.2210/pdb1I1R/pdb] (LIF/gp130 complex). The structure of IL-11Rα used as the initial model for cryo-EM model building is not currently available due to intellectual property considerations. The same analysis of the cryo-EM data can be repeated with the available structure 6O4P [http://doi.org/10.2210/pdb6O4P/pdb].

SAXS data generated in this study and associated atomic model coordinates have been deposited in the Small Angle Scattering Biological Data Bank (SASBDB) with the following accession codes: SASDLM3 [https://www.sasbdb.org/data/SASDLM3/] (IL-11Δ10/IL-11RαD1-D3/gp130D1-D3 complex), SASDLN3 [https://www.sasbdb.org/data/SASDLN3/) (IL-11Δ10/IL-11RαD1-D3/gp130EC complex), SASDLP3 [https://www.sasbdb.org/data/SASDLP3/] (IL-11Δ10/IL-11RαD1-D3/gp130D2-D3 complex), SASDLS3 [https://www.sasbdb.org/data/SASDLS3/] (IL-11Δ10/Mutein/IL-11RαD1-D3/gp130D1-D3 complex), SASDLR3 [https://www.sasbdb.org/data/SASDLR3/] (IL-11Δ10/Mutein), SASDLQ3 [https://www.sasbdb.org/data/SASDLW3/] IL-11Δ10/W147A.

Molecular dynamics protein structure files (PSF) and trajectories have been deposited on Figshare with the following DOIs: 10.6084/m9.figshare.24043518 [https://doi.org/10.6084/m9.figshare.24043518] (IL-11Δ10 microsecond MD trajectory) and 10.6084/m9.figshare.24043527 [https://doi.org/10.6084/m9.figshare.24043527] (IL-11Δ10/Mutein microsecond MD trajectory). Source data for the ITC data presented in Figure 2, Figure 5 and Figure 6 was deposited on Figshare with the following DOI: 10.6084/m9.figshare.24293080[https://doi.org/10.6084/m9.figshare.24293080]. Source data for the SPR data presented in Figure 5D was deposited on Figshare with the following DOI: 10.6084/m9.figshare.24293563[https://doi.org/10.6084/m9.figshare.24293563].

Other data are contained within the manuscript and Supplementary Information. Materials generated in this study may be obtained from the corresponding author for non-commercial research use via a materials transfer agreement. Source data are provided with this paper.

Human research participants

Policy information about studies involving human research participants and Sex and Gender in Research.

Reporting on sex and gender	N/A
Population characteristics	N/A
Recruitment	N/A
Ethics oversight	N/A

Note that full information on the approval of the study protocol must also be provided in the manuscript.

Field-specific reporting

Please select the one below that is the best fit for your research. If you are not sure, read the appropriate sections before making your selection.

☒ Life sciences ☐ Behavioural & social sciences ☐ Ecological, evolutionary & environmental sciences

For a reference copy of the document with all sections, see nature.com/documents/nr-reporting-summary-flat.pdf

Life sciences study design

All studies must disclose on these points even when the disclosure is negative.

Sample size	EM particle sample size was determined by automated particle picking and curation. The particles selected were sufficient to yield density maps with resolutions of 3.5 Å and 3.8 Å, as determined by gold-standard FSC analysis.

	Sample sizes for biochemical and cellular experiments were not predetermined. The sample sizes used were sufficient to represent the reproducibility of the experiments and are in line with conventions in the field.
Data exclusions	Cryo-EM particles providing the highest quality 3D reconstruction were retained. Other particles were excluded. No data were excluded from biochemical and cellular experiments.
Replication	Cryo-EM density maps were calculated from thousands of particles and multiple grids. The cryo-EM structure determination process was not replicated.<br>Three independent experiments were conducted for EC50, IC50, ITC and DSF analyses. Two independent experiments were conducted for analysis of signaling inhibition in cancer cell lines and SPR. Crystal structure determination, SAXS, AUC, SEC-MALS, and MD experiments were not replicated.
Randomization	Not applicable. No organisms or subjects that require randomization were analysed.
Blinding	Not applicable. No organisms or subjects that require blinding were analysed.

Reporting for specific materials, systems and methods

We require information from authors about some types of materials, experimental systems and methods used in many studies. Here, indicate whether each material, system or method listed is relevant to your study. If you are not sure if a list item applies to your research, read the appropriate section before selecting a response.

Materials & experimental systems

n/a	Involved in the study	
☐	☒	Antibodies
☐	☒	Eukaryotic cell lines
☒	☐	Palaeontology and archaeology
☒	☐	Animals and other organisms
☒	☐	Clinical data
☒	☐	Dual use research of concern

Methods

n/a	Involved in the study	
☒	☐	ChIP-seq
☐	☒	Flow cytometry
☒	☐	MRI-based neuroimaging

Antibodies

Antibodies used	Phospho-STAT3 antibody (clone 4/P-STAT3), BD Cat no. 557815<br>Mouse IgG2a, κ Isotype control, Clone MOPC-173 (RUO), BD Cat no. 558053
Validation	QC tested for specificity by manufacturer, BD Biosciences. Positive and negative controls were included in all experiments.

Eukaryotic cell lines

Policy information about cell lines and Sex and Gender in Research

Cell line source(s)	Human gp130/IL-11RA Ba/F3 cells, generated at WEHI (Nandurkar et al., 1996)<br>MDA-MD-231, Cell Bank Australia Code: 92020424<br>A549, Cell Bank Australia Code: 86012804<br>U-87-MG, Cell Bank Australia Code: 89081402<br>BxPC-3, Cell Bank Australia Code: JCRB1448<br>LnCAP, Cell Bank Australia Code: 89110211<br>Spodoptera frugiperda Sf21, Invitrogen Cat no. 11497013
Authentication	Cell lines were purchased, unless indicated otherwise. None of the cell lines were authenticated.
Mycoplasma contamination	Each cell line was routinely tested for mycoplasma and confirmed to be negative prior to experiments.
Commonly misidentified lines (See ICLAC register)	No commonly misidentified lines were used.

Flow Cytometry

Plots

Confirm that:

☒ The axis labels state the marker and fluorochrome used (e.g. CD4-FITC).

☒ The axis scales are clearly visible. Include numbers along axes only for bottom left plot of group (a 'group' is an analysis of identical markers).

☒ All plots are contour plots with outliers or pseudocolor plots.

☒ A numerical value for number of cells or percentage (with statistics) is provided.

Methodology

Sample preparation	Cells were fixed, permeabilised and stained for p-STAT3 (clone: 4/P-STAT3, BD Biosciences).
Instrument	LSR II SORP, BD
Software	FloJo 10.8.2
Cell population abundance	Not applicable. Cell sorting experiments were not performed.
Gating strategy	Cells were gated on live single cells and percentage with positive staining indicated.

☒ Tick this box to confirm that a figure exemplifying the gating strategy is provided in the Supplementary Information.
